# Neurological manifestations and genotype–phenotype correlations in *NDUFAF6*-associated mitochondrial disease

**DOI:** 10.1093/braincomms/fcag095

**Published:** 2026-03-18

**Authors:** Alessandra Torraco, Charlotte L Alston, Giulia Barcia, Daniela Verrigni, Teresa Rizza, Michela Di Nottia, Anastasia Altobelli, Diego Martinelli, Daria Diodato, Stephanie Efthymiou, Melis Kose, Yamna Kriouile, Albert Z Lim, Silvia Morlino, Barbara Siri, Nebal Waill Saadi, Antonio Novelli, Henry Houlden, Carlo Dionisi-Vici, Robert McFarland, Agnès Rötig, Enrico Bertini, Robert W Taylor, Rosalba Carrozzo

**Affiliations:** Laboratory of Medical Genetics, Translational Cytogenomics Research Unit, Bambino Gesù Children's Hospital, IRCCS, 00146 Rome, Italy; Mitochondrial Research Group, Translational and Clinical Research Institute, Faculty of Medical Sciences, Newcastle University, Newcastle upon Tyne NE2 4HH, UK; NHS Highly Specialised Service for Rare Mitochondrial Disorders, Newcastle upon Tyne Hospitals NHS Foundation Trust, Newcastle upon Tyne NE1 4LP, UK; Service de Médecine Génomique des Maladies Rares—APHP, Hôpital Necker Enfants Malades, Imagine Institute, Université Paris Cité, 75015 Paris, France; Laboratory of Medical Genetics, Translational Cytogenomics Research Unit, Bambino Gesù Children's Hospital, IRCCS, 00146 Rome, Italy; Laboratory of Medical Genetics, Translational Cytogenomics Research Unit, Bambino Gesù Children's Hospital, IRCCS, 00146 Rome, Italy; Laboratory of Medical Genetics, Translational Cytogenomics Research Unit, Bambino Gesù Children's Hospital, IRCCS, 00146 Rome, Italy; Laboratory of Medical Genetics, Translational Cytogenomics Research Unit, Bambino Gesù Children's Hospital, IRCCS, 00146 Rome, Italy; Division of Metabolic Diseases and Hepatology, Bambino Gesù Children's Hospital, IRCCS, 00165 Rome, Italy; Neuromuscular Disorders Research Unit, Bambino Gesù Children's Hospital, IRCCS, 00146 Rome, Italy; Department of Neuromuscular Diseases, UCL Queen Square Institute of Neurology, Queen Square WC1N 3BG London, UK; Division of Human Genetics, Department of Pediatrics, The Children's Hospital of Philadelphia, Philadelphia, PA 19104, USA; Unit of Neuropediatrics and Neurometabolism, Pediatric Department 2, Rabat Children's Hospital, BP 6527 Rabat, Morocco; Mitochondrial Research Group, Translational and Clinical Research Institute, Faculty of Medical Sciences, Newcastle University, Newcastle upon Tyne NE2 4HH, UK; NHS Highly Specialised Service for Rare Mitochondrial Disorders, Newcastle upon Tyne Hospitals NHS Foundation Trust, Newcastle upon Tyne NE1 4LP, UK; Division of Medical Genetics, Fondazione IRCCS-Casa Sollievo della Sofferenza, 71013 San Giovanni Rotondo, Italy; Division of Metabolic Diseases and Hepatology, Bambino Gesù Children's Hospital, IRCCS, 00165 Rome, Italy; Unit of Pediatric Neurology, College of Medicine/University of Baghdad, Children Welfare Teaching Hospital, 12114 Baghdad, Iraq; Laboratory of Medical Genetics, Translational Cytogenomics Research Unit, Bambino Gesù Children's Hospital, IRCCS, 00146 Rome, Italy; Department of Neuromuscular Diseases, UCL Queen Square Institute of Neurology, Queen Square WC1N 3BG London, UK; Division of Metabolic Diseases and Hepatology, Bambino Gesù Children's Hospital, IRCCS, 00165 Rome, Italy; Mitochondrial Research Group, Translational and Clinical Research Institute, Faculty of Medical Sciences, Newcastle University, Newcastle upon Tyne NE2 4HH, UK; NHS Highly Specialised Service for Rare Mitochondrial Disorders, Newcastle upon Tyne Hospitals NHS Foundation Trust, Newcastle upon Tyne NE1 4LP, UK; Institut Imagine, Génétique des maladies mitochondriales, INSERM UMR 1163, Université Paris Cité, 75015 Paris, France; Neuromuscular Disorders Research Unit, Bambino Gesù Children's Hospital, IRCCS, 00146 Rome, Italy; Mitochondrial Research Group, Translational and Clinical Research Institute, Faculty of Medical Sciences, Newcastle University, Newcastle upon Tyne NE2 4HH, UK; NHS Highly Specialised Service for Rare Mitochondrial Disorders, Newcastle upon Tyne Hospitals NHS Foundation Trust, Newcastle upon Tyne NE1 4LP, UK; Laboratory of Medical Genetics, Translational Cytogenomics Research Unit, Bambino Gesù Children's Hospital, IRCCS, 00146 Rome, Italy

**Keywords:** Leigh syndrome, Mitochondrial disease, Respiratory chain complexes, NADH–ubiquinone oxidoreductase, Assembly factors

## Abstract

*NDUFAF6* encodes a mitochondrial complex I assembly factor essential for the proper biogenesis and stability of the nicotinamide adenine dinucleotide (NAD) + hydrogen (H) (NADH)–ubiquinone oxidoreductase complex. Pathogenic variants in *NDUFAF6* have been increasingly recognized as a cause of mitochondrial disease, particularly Leigh syndrome, a severe neurodegenerative disorder characterized by bilateral symmetrical lesions in the central nervous system. To date, fewer than 50 patients with *NDUFAF6*-related mitochondrial disease have been reported, displaying a broad phenotypic spectrum ranging from early-onset neurodevelopmental regression to milder, more chronic presentations. The molecular mechanisms underlying these phenotypes are linked to impaired complex I assembly and reduced enzymatic activity, highlighting the critical role of NDUFAF6 in mitochondrial function.

Here we present a cohort of 27 patients (14 males and 13 females) from 18 families harbouring biallelic variants in the *NDUFAF6* gene. The patient’s mean age was 9.15 ± 8.30 years (range: 4 weeks to 25 years); 12 patients (37%) had died by the time the data were collected for this article. The clinical presentation showed wide phenotypic variability, from mild to severe psychomotor regression (74%) most commonly before the age of 5 years, hypotonia (22%), movement disorders (30%), and hypertonia (15%). Bilateral striatal necrosis lesions were the most characteristic features on cranial MRI (67%) although white matter abnormalities were also noted (15%), occasionally accompanied by cystic formations, suggestive of early neurodevelopmental anomalies.

Genomic sequencing was applied, leading to the identification of 19 distinct variants in the *NDUFAF6* gene, including nine novel variants not previously reported and either absent or extremely rare in public population databases. Functional studies confirmed the pathogenicity of these variants, demonstrating a deleterious effect on NDUFAF6 protein expression and a consequent impairment in complex I assembly and stability.

To date, this represents the largest reported cohort of patients with *NDUFAF6*-associated mitochondrial disease. Our findings provide a comprehensive overview of clinical characteristics—including age of symptom onset, phenotypic variability, and patient outcomes—aiming to improve prognostic information and facilitate genetic counselling in clinical practice.

## Teaser

Torraco *et al.* report that variants in *NDUFAF6* cause a heterogeneous mitochondrial disease characterized primarily by bilateral striatal necrosis and reduced complex I activity. Functional studies confirmed the pathogenicity of novel variants, thereby expanding the phenotypic spectrum and highlighting the relevance of personalized genetic counseling and prognostic assessment in clinical practice.

## Graphical Abstract

**Graphical Abstract:**
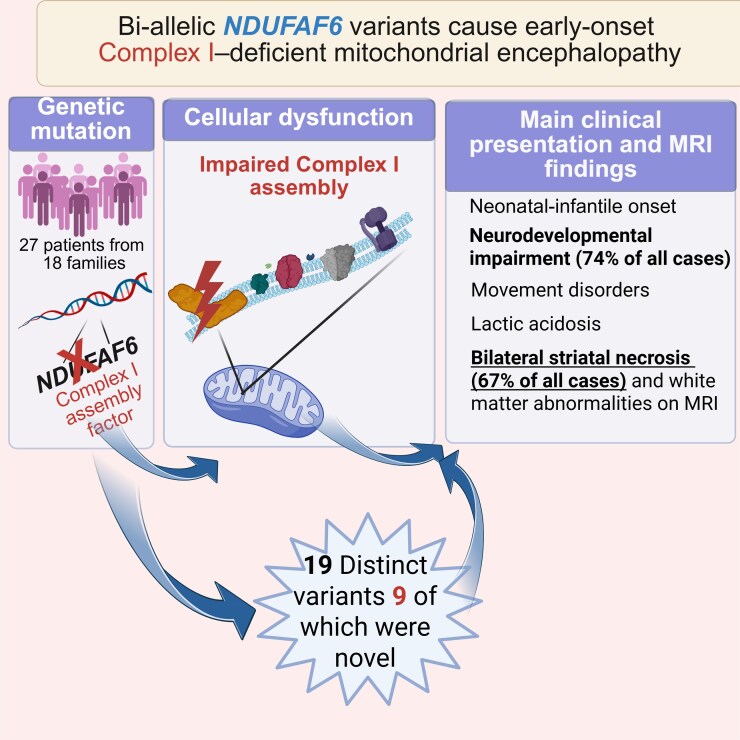

## Introduction

Complex I (CI) or NADH–ubiquinone oxidoreductase provides the entry point for the electrons coming from NADH oxidation into the respiratory chain. Mammalian CI is the product of both nuclear (nDNA) and mitochondrial (mtDNA) genomes and is composed of 44 subunits^1^ with an estimated size of about 1 MDa.^2^ It has a unique L-shaped structure with a hydrophilic arm protruding into the mitochondrial matrix and a hydrophobic arm embedded within the inner mitochondrial membrane. Of the 44 subunits, 14 (seven mtDNA and seven nDNA encoded subunits) constitute the catalytic core of the enzyme and are all located in the membrane arm; the remaining nDNA encoded proteins, defined as ‘supernumerary’, shield the metal centres of the enzyme and have roles in CI biogenesis and stability. From a functional point of view, we can distinguish three modules: the NADH dehydrogenase module (N-module), the proton translocating module (*P*-module), and the ubiquinone-binding module (Q-module). Due to its complexity and the large number of subunits, the construction of CI is an intricate stepwise process that requires the involvement of numerous assembly factors that facilitate the insertion of the structural subunits in an orchestrated manner.^3^ To date, almost 20 assembly factors are known to be involved in the construction of CI, but the number is set to increase.^4-8^

Complex I deficiency (MIM: 252010) is the most common defect found in patients with mitochondrial disease^9^ and is associated with a wide variety of clinical phenotypes ranging from the most severe infantile onset disease (i.e. Leigh syndrome, LS; fatal infantile lactic acidosis; leukodystrophy; hypertrophic cardiomyopathy; hepatopathy) to milder forms of disease with indolent myopathy or deafness in adults^10^ or to optic neuropathies, both maternally and autosomally inherited, including Leber hereditary optic neuropathy (LHON).^11-13^ Variants have been found both in CI structural subunits, encoded by mitochondrial DNA (mtDNA) and nuclear DNA (nDNA), and in numerous assembly chaperones.

Leigh syndrome (MIM 25600) is the most common paediatric mitochondrial disease presentation, with an estimated prevalence of 1:40 000.^14-16^ It is a progressive neurodegenerative disorder characterized by symmetrical lesions in the thalamus, brainstem, and posterior columns of the spinal cord. Typically, the primary symptoms of Leigh syndrome—dystonia–spasticity, dysphagia, hypopnoea, lactic acidosis, and neurodevelopmental regression—become increasingly prominent during the second and third years of life, frequently in the context of an acute or subacute metabolic crisis. In contrast, secondary symptoms including vomiting, failure to thrive, and mild developmental delay often appear earlier, before 1 year of age, but are less specific indicators of the disorder. Death may result from aspiration pneumonia or central respiratory failure (apnoea) during these acute deteriorations. Multisystemic presentations may also occur including cardiac, gastrointestinal, and/or renal tubular defects. Some patients who develop LS during adolescence or adulthood have a milder disease course and better prognosis.^17^ More than 30% of patients with LS have a biochemical defect involving CI,^16,18^ but a large proportion of patients—particularly those investigated prior to the mainstream integration of diagnostic next generation sequencing—lack a molecular diagnosis.

Here we report a group of patients affected by LS who harbour biallelic pathogenic variants in *NDUFAF6*, a gene encoding an assembly factor of CI. To our knowledge, this report represents the largest group of patients with *NDUFAF6-*associated mitochondrial disease. This study aims to provide an overview of the cohort characteristics including the age of symptom onset, the phenotypic spectrum, and the patient outcomes due to defects involving the *NDUFAF6* gene, with a view to provide prognostic information to facilitate improved family counselling in clinical practice.

## Materials and methods

### Study design

The 27 patients were recruited from several diagnostic and research genetic laboratories worldwide. Inclusion criteria were (i) carrying biallelic pathogenic or likely pathogenic variants in *NDUFAF6* gene and (ii) having a complete clinical assessment at respective centres.

The study was approved by the Ethical Committees of the Bambino Gesù Children’s Hospital (Rome, Italy), the Necker Hospital (Paris, France), UCL Queen Square Institute of Neurology (London, UK), and the Newcastle upon Tyne Hospitals NHS Foundation Trust (Newcastle upon Tyne, UK) in agreement with the Declaration of Helsinki. Informed consent for molecular genetic analysis and to participate in the study was obtained from all patients or their parents in case of paediatric patients. The manuscript reflects a research activity conducted in compliance with the Hospital Code of Ethics and Internal Regulations.

### Genetic analysis

DNA from patients and family members was isolated from EDTA–blood samples and/or primary skin fibroblasts using standard procedures. Different next generation sequencing approaches in various laboratories have been used. In detail, P1, P3, and P5 were sequenced using a clinical exome panel (TruSight One Expanded Sequencing Panel),^19^ and Sanger sequencing was applied for P2 and P4; P6-13 were analysed with a sequencing panel targeting 380 nuclear genes associated with mitochondrial disease;^20^ P14-26 were tested either by targeted NGS using a bespoke Ampliseq capture focusing on CI structural subunits and assembly factors^21,22^ or gene agnostic WES, as described elsewhere;^23^ P27 underwent clinical genetic testing at CENTOGENE GmbH.^24^ Bioinformatic analysis was performed using different approaches, and reads were aligned to either the GRCh37/hg19 or GRCh38/hg38 genome assembly. For all patients, *NDUFAF6* variants were annotated according to HGVS variant nomenclature guidelines (http://varnomen.hgvs.org/),^25^ using the reference sequence RefSeq NM_152416.4; variant classification was performed according to ACGS (https://www.genomicseducation.hee.nhs.uk/wp-content/uploads/2024/08/ACGS-2024_UK-practice-guidelines-for-variant-classification.pdf) and ACMG/AMP classification guidelines.^26^ Segregation and validation of the variants in available parents and siblings was undertaken using Sanger sequencing.

### RNA extraction and cDNA studies

cDNA studies in P16 were undertaken using RNA derived from patient and control fibroblasts. RNA extraction was performed using the Trizol reagent, and the reverse transcription was carried out using the MMLV reverse transcriptase (Promega) according the manufacturers’ protocol. PCR primers were designed to span exon–exon boundaries, and resultant amplicons were subject to Sanger sequencing to determine the consequence of the patient’s putative splicing variant on mRNA splicing.

### Cell culture and mitochondria isolation

Patients fibroblasts were obtained from skin biopsy and cultured in DMEM media (4.5 g/L glucose), supplemented with 10% foetal bovine serum and 50 μg/ml uridine. Mitochondria were isolated from cultured skin fibroblasts as previously described.^27^

### Protein studies

For P1, P2, P3, P5, P6, and P8, SDS–PAGE was performed loading 35–100 μg of mitochondria on a 4–12% Bis–Tris precast polyacrylamide gel (Thermo Fisher Scientific). Proteins were transferred using an Invitrolon™ PVDF filter paper sandwich (0.45μm pore size, Thermo Fisher Scientific) and probed with specific antibodies. For Blue native gel electrophoresis (BNGE) studies, mitochondrial pellets derived from the same patient cell lines were processed using previously described methods^28^ and gels subjected to either Western blotting using specific antibodies or to in situ activity assay, as described elsewhere.^29^

### Antibodies

Immunodetection of specific subunits of the respiratory chain complexes was achieved using the following antibodies: CI-NDUFA9 and NDUFS3 (1:1000, ab14713 and ab14711, Abcam); NDUFS1 (1:1000, sc-50131, Santa Cruz), NDUFB11 (1:1000, 16720-1-AP, Proteintech), and NDUFAF6 (1:500, HPA047148, Sigma-Aldrich); complex II-SDHA (1:1000, ab14715, Abcam), complex III-UQCRC2/CORE2 (1:1000, ab14745, Abcam), complex IV-COXI (1:1000, PA5-26688, Thermo Fisher Scientific), and COX2 (1:1000, ab110258, Abcam); VDAC (1:1000, 55259-1-AP, Proteintech); and Vinculin (1:1000, V9131, Sigma-Aldrich). Reactive bands were detected using Clarity western ECL Substrate (BioRad). Densitometry analysis was performed using Quantity One software (BioRad). Protein concentration was determined by BCA assay (Thermo Fisher Scientific).

### Statistical analysis

Statistical analysis was performed using Student’s *t*-test. A *P*-value < 0.05 (*) was considered statistically significant, *P* < 0.005 (**) was considered highly significant, and *P* < 0.0005 (***) was considered extremely significant.

### Literature review

For the comparative analysis with published data, we used the online databases PubMed and Google Scholar. The search terms employed were ‘NDUFAF6’ and ‘C8ORF38’. We extracted only the key clinical features and MRI findings from the reported cases to facilitate comparison with our cohort. Articles reporting large cohorts of CI-deficient patients without detailed individual clinical information were excluded. No statistical analysis was undertaken.

## Results

### Clinical findings

**Figure 1:**
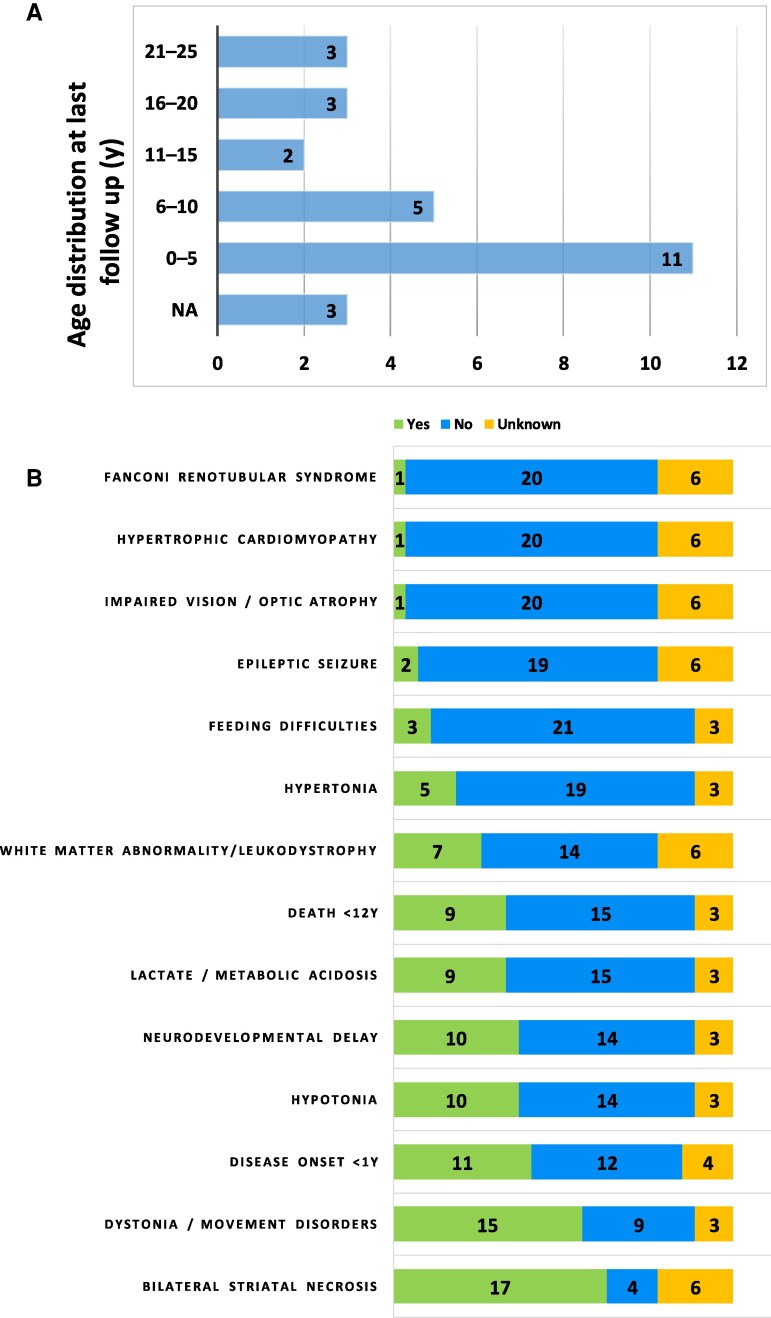
**Distribution of clinical and neuroradiological data.** (**A**) Distribution of patients’ age at last follow-up. (**B**) Clinical features of the affected individuals with biallelic *NDUFAF6* variants. The bar chart illustrates the most common clinical symptoms and signs of our patients alongside the key neuroradiological findings. The x-axis represents the number of patients (n = 27), while the y-axis lists the main clinical features and radiological findings observed. The green bars indicate the number of patients exhibiting each specific feature; the blue bars represent patients without that feature; and the orange bars correspond to cases with missing clinical information.

We collected a total of 27 patients (14 male, 13 female) from 18 previously unreported families affected by LS and carrying biallelic pathogenic or likely pathogenic (P/LP) variants in the *NDUFAF6* gene. Detailed clinical information of our patient cohort is provided in the supplementary results. The main features of the disease course, neuroimaging, and genetic findings are listed in Fig. 1, Supplementary Table 1 and Supplementary Table 2. Overall, the mean age of the patients was 9.15 ± 8.30 years (range: 4 weeks to 25 years), and no sex differences were observed. Twelve patients died and for 10 of them this occurred before 12 years. The disease onset typically occurred in early childhood and exhibited a markedly variable clinical course. While some patients (P2, P10, P11, P17, P19, P22) died within the first year of life, others (P1, P7, P8, P9, P13, P14) survived into later years, with some reaching adulthood and remaining clinically stable. About half of the families were consanguineous. Clinical symptoms at onset were variable, including psychomotor delay, hypotonia, dystonia, feeding difficulties, lactic acidosis, and epileptic seizures. One patient developed Fanconi syndrome (P1). Brain imaging, when available, displayed neuroimaging features of Leigh syndrome in most of the patients. In P1, a brain MRI at 24 months showed alteration of the white matter with pseudo cystic appearance and involvement of the corpus callosum. The lesions remained stable until 14 years of age when a new MRI displayed a progression of the disease (Supplementary Fig. 1).

### Molecular findings

**Figure 2:**
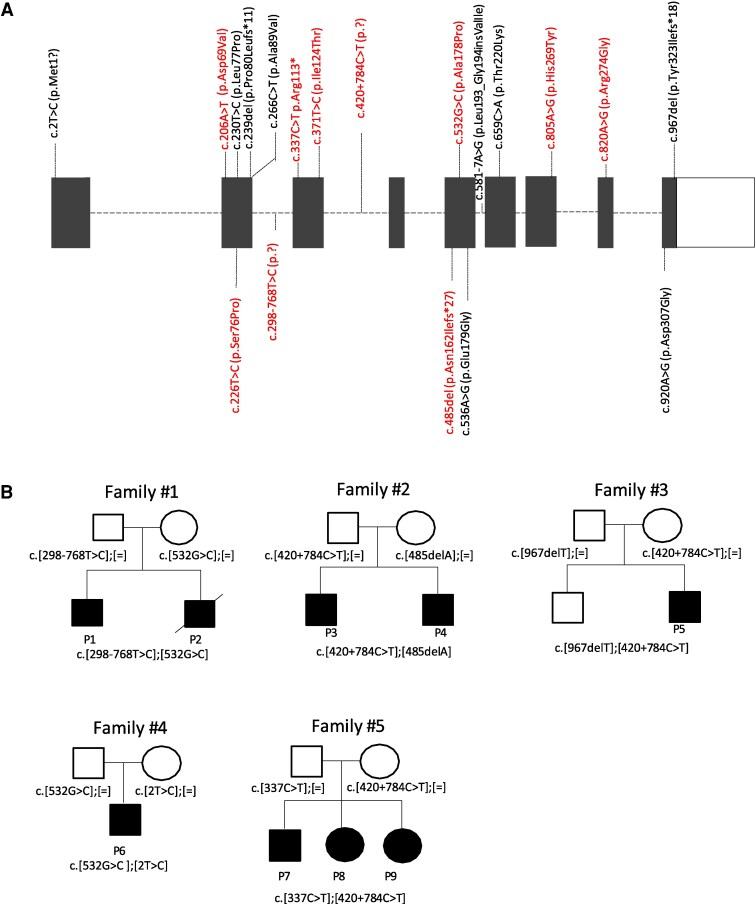
**Distribution of *NDUFAF6* variants and pedigrees.** (**A**) Schematic representation of *NDUFAF6* gene showing the position of the variants. Variants found in our patients and already described in the literature are shown in red, whilst the novel variants are shown in black. (**B**) Pedigrees showing the *NDUFAF6* variant segregation for patients who have undergone functional analysis.

A schematic representation of all the variants described in this report is shown in Fig. 2A, whereas the pedigrees annotated with variants are shown only for the patients where functional studies have been performed (Fig. 2B).

**Figure 3:**
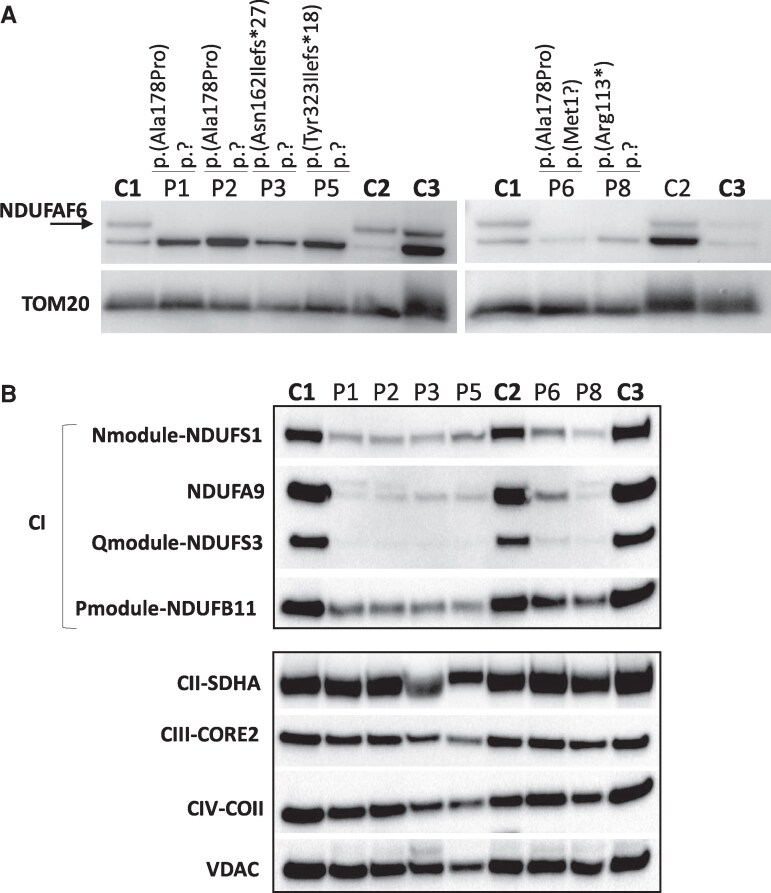
**NDUFAF6 expression and steady state level of the RC complexes subunits.** (**A**) SDS–PAGE performed on mitochondria isolated from skin fibroblasts of the patients (P1, P2, P3, P5, P6, P8) and age matched controls (C1, C2, C3). The uncropped blot is provided as Supplementary File 1 in the Supplementary Materials section. (**B**) Skin fibroblast-derived mitochondria were isolated from patients (P1, P2, P3, P5, P6, P8) and controls (C1, C2, C3) and loaded on a SDS gel. Specific antibodies were used to detect CI subunits (NDUFS1, NDUFA9, NDUFS3, and NDUFB11), CII subunit (SDHA), CIII subunit (CORE2), and CIV subunit (COXII). The uncropped blots are provided as Supplementary Files 2 and 3 in the Supplementary Materials section. CI, complex **I**, CII, complex II; CIII, complex III; CIV, complex IV.

**Figure 4:**
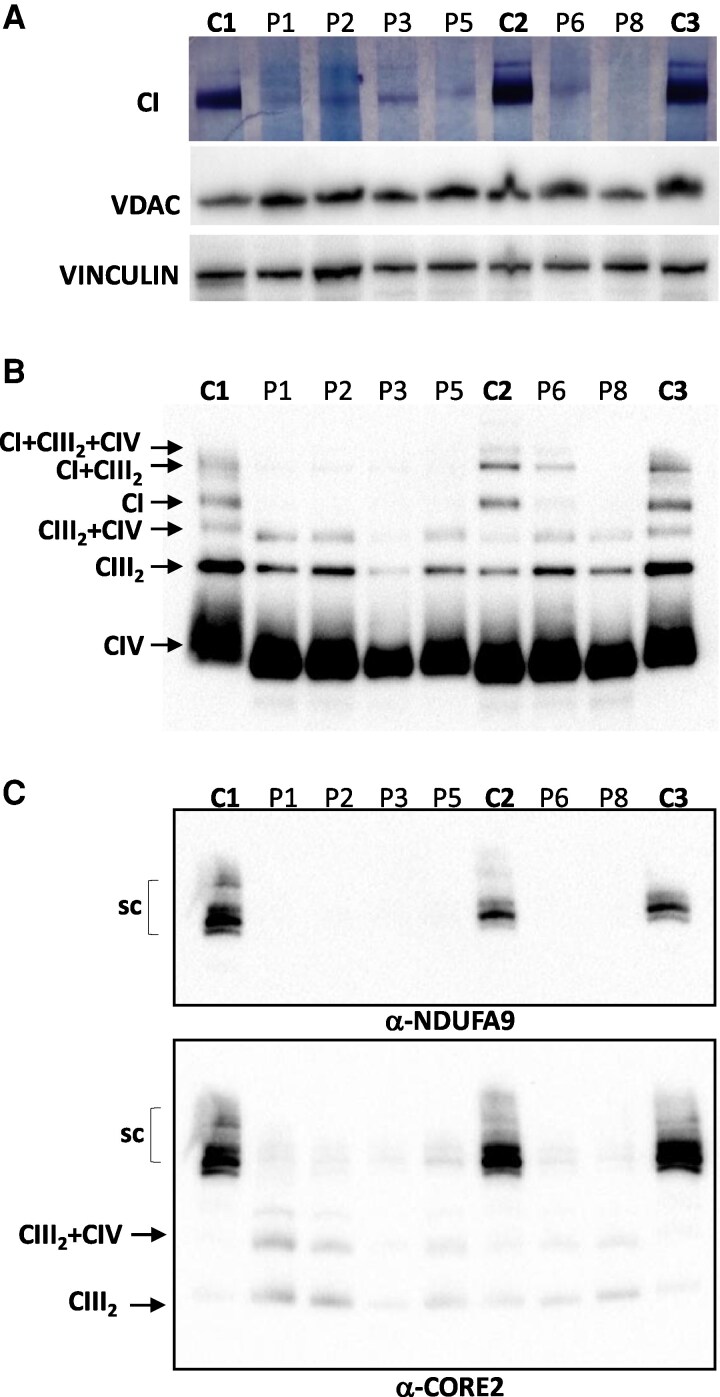
**Assembly status of complex I.** BNGE of DDM treated mitochondria were subjected to (**A**) *in-gel* activity assay of CI and (**B**) Western blotting analysis using specific antibodies conjugated against subunits of CI (NDUFA9), CIII (CORE2), and CIV (COXI). VDAC and Vinculin were used as loading controls (A and B). (**C**) Digitonin treated mitochondria were subject to BNGE, separated on 3–12% non-denaturing gels to resolve mitochondrial supercomplexes (SC). Western blotting was performed using specific antibodies to detect CI- and CIII-containing SC (NDUFA9 and CORE2, respectively). The uncropped blots are provided as Supplementary Files 4 and 5 in the Supplementary Materials section. CI, complex I; CII, complex II; CIII, complex III; CIV, complex IV. BNGE, Blue native gel electrophoresis; DDM, N-dodecyl-beta-D-maltoside; VDAC, voltage-dependent anion channel.

Among the 27 individuals of our cohort, we found a total of 19 different heterozygous *NDUFAF6* variants (NM_152416.4) distributed as follows: 11 missense, 3 intronic/splicing, 3 frameshift, 1 nonsense, and 1 variant that abolished the initiation methionine. The variants were located throughout the gene and were not predicted to cluster around a specific region or domain of the protein (Fig. 2A). Two of the 19 variants, the c.532G > C (p.Ala178Pro) missense variant and the c.420 + 784C > T deep intronic variant, have been reported previously in the literature^30^ and have been found in several families within our cohort of patients (family 1, 4, 6, 9, and 15 and family 2, 3, 5, and 9, respectively) (Supplementary Tables 1 and 2). This suggests that these variants may originate from an ancient founder event in Mediterranean populations, although genetic linkage data are currently lacking to confirm this. Of the 17 remaining variants, eight have been already reported in the literature,^31-34^ and nine were novel and either absent or present at a very low frequency in the Genome Aggregation Database (gnomAD v2.1.1 or v4.1.0) (Supplementary Table 2). The deep intronic variants c.420 + 784C > T and c.298-768T > C have been already reported,^30,31^ and both have been proven to cause nonsense-mediated decay of the associated *NDUFAF6* mRNA transcript. Regarding the third intronic variant, c.581-7A > G, found in P16, our cDNA studies using patient fibroblast-derived RNA confirmed that the variant introduces a cryptic acceptor site with a consequent addition of two amino acid residues into the NDUFAF6 protein, p.(Leu193_Gly194insValIle) (Supplementary Fig. 2A and B). All the *NDUFAF6* variants segregated with disease in the family, with healthy parents carrying a single heterozygous variant. Only for P6, carrying the c.2T > C and c.532G > C variants, for P12 carrying the homozygous c.266C > T variant, and for P16, carrying the c.581-7A > G and the c.805C > T, segregation analysis was incomplete because of the absence of the father; nevertheless, a selected reduction of CI content was evident in P6 and P16, and biochemical evaluation of CI activity was reduced in patient fibroblasts of P6 (Fig. 3B, Supplementary Fig. 3B, Fig. 4A  **and Supplementary Results**), pointing towards a deleterious contribution of these variants on CI function. All missense variants are predicted to meet the clinically actionable threshold of pathogenic or likely pathogenic according to the ACGS and ACMG/AMP recommendations (Supplementary Table 2). Interestingly, a recent study applied a deep mutational scanning (DMS) approach^35^ to systematically test the pathogenicity of almost all missense variants in *NDUFAF6*, supporting the deleterious nature of the majority of the missense variants identified within this study. Notably, 10 of the 11 *NDUFAF6* missense variants found within our cohort (p.Ala178Pro, p.Glu179Gly, p.Asp307Gly, p.Ala89Val, p.Ser76Pro, p.His269Tyr, p.Thr220Lys, p.Asp69Val, p.Arg274Gly, p.Ile124Thr) were reported to have a deleterious impact on CI activity/stability.^36^ The p.(Leu77Pro) *NDUFAF6* variant was not tested using the DMS approach.

### Effects of *NDUFAF6* variants on RC complexes stability

To investigate the effect of the variants on NDUFAF6 stability, we performed SDS–PAGE and Western blotting analysis on mitochondria isolated from available fibroblasts of patients (P1, P2, P3, P5, P6, and P8) and controls and found an undetectable amount of NDUFAF6 protein (Fig. 3A). As expected, the drastic decrease of NDUFAF6 protein was paralleled by a decrease of several CI subunits as seen by Western blotting using specific antibodies (Fig. 3B upper panel), whereas the subunits of the other RC complexes were expressed within the control range (Fig. 3B lower panel). Of note, not all CI subunits exhibited the same degree of reduction following NDUFAF6 depletion within the same patient. In particular we noticed a strong reduction of NDUFS3, belonging to the Q-module of CI, and a reduction of NDUFA9, a subunit whose module assignment is still controversial^37^ but that is supposed to be inserted in the Q module at a late stage^38^ that was decreased but to a lesser extend compared with NDUFS3. The N-module, represented by NDUFS1, was even less affected than the Q-module, and, finally, the membrane embedded *P*-module NDUFB11-subunit was more stable (Fig. 3B upper panel).

Subsequently, we looked at the assembly state and activity of the RC complexes by BNGE and processed the gels either for in gel activity assay or for Western blotting. All patients showed a dramatic reduction of CI activity when compared with control subjects (Fig. 4A), although after longer incubation with CI substrates a residual activity of CI was evident in all patients underlining that some residual CI could still form and be functional. This result was further supported by the Western blotting of the BNGE that confirmed the reduction of CI in all tested patients (Fig. 4B). No subassemblies were observed in the patients using NDUFA9 antibody. Blue native gel electrophoresis performed for P11, P13, P14, P16, P17, and P19 showed a selected reduction of CI as well (Supplementary Fig. 3A and B).

To evaluate the impact of NDUFAF6 reduction on supercomplexes (SC) stability, we separated digitonin solubilized mitochondrial proteins on 3–12% non-denaturing gels and probed with antibodies targeting specific subunits of CI and CIII. In accordance with the key role of NDUFAF6 in CI assembly, we found no signal corresponding to free CI and a dramatic reduction of the CI-containing SC in patient samples compared with controls (Fig. 4C). In addition, and as expected, the largest amount of CIII was present in its free form or associated to CIV (CIII2 + CIV) and not assembled into the SC containing CI (Fig. 4C).

### Overview of the current literature

We conducted a comparative analysis of our patient cohort alongside previously published cases carrying mutations in the *NDUFAF6* gene (Supplementary Table 3). Consistent with the clinical features and MRI findings observed in our patients, the majority of reported cases presented with clinical features of Leigh syndrome (n = 14), typically associated with characteristic basal ganglia lesions on MRI, predominantly involving the putamen (14 of 14) and, to a lesser extent, the caudate nucleus (6 of 14). Nine patients carrying the homozygous deep intronic variant *c.298-768T*  *>*  *C* exhibited a renal phenotype consistent with Fanconi syndrome.^31^ The average age of disease onset in the published reports was 28.7 months (range: 7 months to 5 years), in line with our cohort. At the time of publication, patients with Leigh syndrome ranged in age from 22 months to 11 years. Only one patient was followed into adulthood, reaching 27 years of age (7%).^39^ In our cohort, approximately 22% of patients survived beyond 18 years, maintaining a relatively stable clinical course. Movement disorders were among the most prominent features in patients with Leigh syndrome. Generalized dystonia was reported in 71% of published cases, with onset typically occurring between 4 and 11 years of age (median: 5.4 years). In our cohort, generalized dystonia was present in 41% of cases, with a similarly early onset consistent with the literature (range: 14 months to 10 years; median: 3 years). Finally, similar to what we observed, the majority of patients reported in previously published studies had decreased CI activity.

## Discussion

Here we report on a cohort of 27 patients harbouring disease-causing *NDUFAF6* variants from 18 families, with the majority showing a common neuroradiological hallmark characterized by symmetrical lesions of the basal ganglia, compatible with Leigh syndrome. Clinically, most of patients presented with psychomotor delay, followed by progressive decline in motor and cognitive abilities. In several families (#2: P3 and P4; #5: P7, P8, and P9; #10: P14 and P15), movement disorders such as dystonia, bradykinesia, and spastic–dystonic tetraparesis were present, often accompanied by severe dysarthria and eventual loss of ambulation, frequently leading to wheelchair dependence. Epilepsy was observed only in two patients (#7: P11; #17: P26), whereas optic nerve atrophy in three (#15: P23; #16: P24, P25). Only one patient developed renal Fanconi syndrome (#1: P1). An extensive review of the literature on patients carrying biallelic variants in the *NDUFAF6* gene confirmed several distinctive features, including the symmetrical basal ganglia lesions characteristic of LS and the frequent emergence of extrapyramidal movement disorders. Consistent with our cohort, disease onset in published cases typically occurred in early childhood, although the clinical course was highly variable. While some patients died early, often following an acute metabolic crisis, others survived into adolescence or adulthood with a relatively stable disease course. Importantly, isolated CI deficiency was reported in nearly all published cases, regardless of the specific *NDUFAF6* variants identified. Our patients harboured biallelic variants in the *NDUFAF6* gene, encoding a mitochondrial CI assembly factor. Next-generation sequencing identified 19 rare variants, including 10 previously reported and several novel or ultra-rare alleles. NDUFAF6 expresses multiple transcripts,^31,40^ but only the canonical isoform (NM_152416.4) encodes a mitochondrial-targeted protein essential for early CI assembly.^40^ Pathogenic variants result in defective CI biogenesis. Blue native gel electrophoresis demonstrated isolated reductions in CI steady-state levels and enzymatic activity across all patients, without complete loss, indicating residual functional CI. This residual activity may underlie phenotypic variability. CI deficiency also led to significant respirasome (CI/III_2_/IV) depletion and accumulation of homodimeric complex III, consistent with previously characterized CI assembly defects.^41,42^ The decrease in fully assembled CI correlated with a reduction of individual CI subunits, though the extent varied by module. Complex I biogenesis is a stepwise process where membrane arm subunits (NDs and accessory proteins) form the P module, critical for full CI assembly, while remaining accessory subunits integrate into matrix-associated Q and N modules. Interestingly, upon inactivation of NDUFAF6, we found a dramatic reduction of NDUFS3 belonging to the Q module, an intermediate reduction of NDUFS1 belonging to the N module and an even milder decrease of NDUFB11 belonging to the P module. Previous studies have shown that upon CI defect, the turnover of the subunits varies greatly by module, with the Q module being most sensitive to degradation, the N module that accumulates longer, and the P module that is most stable.^37,38,43^ Recent studies have shown that NDUFAF6 interacts with NDUFS8, a core subunit belonging to the Q module and that the ablation of NDUFAF6 prevents the incorporation of NDUFS8 into the Q module, thus inhibiting its maturation.^36^ Interestingly, our results overlap with those recently reported,^36^ even to the extent of the variable expression of CI subunits determined by the variable influence of *NDUFAF6* variants on NDUFAF6-NDUFS8 interaction and therefore Q module formation. Taking these findings together, our results support the hypothesis that residual levels of CI subunits are sufficient to permit the formation of a small amount of functionally active CI, which is subsequently able to assemble into SC. The impact that residual CI has on the disease course is difficult to assess, although for patients that share a severe bioenergetics defect involving CI their disease course was often dramatically disparate. Even in the context of the same family, a highly variable clinical course occurred. Indeed, in family #1, two siblings had an early onset of the disease but with a totally different outcome. P1 developed an early metabolic decompensation which evolved into multisystem involvement with a stable, normal neurological examination; he presented with acute hemiparesis with brain MRI lesions in the early stage of the disease; anyway, the lesions remained quite stable over time and the hemiparesis recovered completely. Conversely, his younger brother P2 also had a metabolic crisis from which he did not recover and subsequently died. Interestingly, P1 developed renal Fanconi syndrome and carried the same deep intronic *NDUFAF6* variant described in a homozygous state in the Acadian population and associated with the same clinical condition.^31^ Several mitochondrial diseases have been reported to be associated with renal Fanconi syndrome (a proximal tubule dysfunction, due to impaired energy metabolism, causing loss of various solutes in urine), such as Kearns–Sayre syndrome, mtDNA depletion syndromes caused by pathogenic variants in nuclear genes involved in mtDNA maintenance (e.g. *DGUOK*, *TK2*), or isolated CI deficiency (caused by variants in CI assembly factors; e.g. *NDUFAF6*, *NDUFAF4*). It is difficult to assess if the c.298-768T > C variant might be causative of the renal phenotype in our patient since heterozygous patients carrying this specific variant were healthy. The early death of P1’s younger affected brother (#1: P2) prevents us from determining whether he would have developed the same renal phenotype. Nevertheless, it is tempting to speculate that a more deleterious variant, such as c.532G > C (p.Ala178Pro), might modulate the renal penetrance of the c.298-768T > C variant. This marked intrafamilial phenotypic variability suggests the involvement of additional modifying factors, which may include environmental influences, epigenetic modifications, or other genetic variants acting as modifiers. It is also difficult to interpret the phenotypic spectrum associated with the same variants in different patients, although modifying environmental factors or additional genetic variants may be involved, particularly for patients belonging to the same family with a shared environment. It is tempting to speculate that either low levels of fully assembled CI, when sustained by certain drugs, may be sufficient to maintain subnormal CI activity that still adequately meets the energy demands of specific tissues or that the presence of functional collateral pathways might be activated or enhanced by the early administration of vitamins and supplements.

Although establishing a prognosis for patients with mitochondrial disease is challenging due to the wide variety of genotypes and phenotypes and the often poor genotype–phenotype correlation, it remains crucial to continue correlating clinical phenotypes with specific genotypes, as this can, in some cases, enhance genetic counselling and improve predictions of disease progression. One of the largest mitochondrial disease natural history studies consisted of a group of 131 patients with different clinical features and genetic diagnoses.^44,45^ Amongst this group, those patients who had cardiomyopathy died earlier, whereas patients with Leigh syndrome without cardiomyopathy lived longer and may also have a more stable course of the disease.

Overall, our data support the importance of widening the phenotype–genotype descriptions, because even though the clinical picture can be non-specific for a disease gene, detailed and attentive phenotype description, segregation information along the family members, and targeted functional analyses are fundamental to assess the pathogenicity of new variants, predict the clinical prognosis of the disease, and improve the care of these patients through informed genetic counselling.

Future studies should aim to identify potential genetic modifiers and environmental factors that contribute to the phenotypic variability observed in *NDUFAF6*-associated mitochondrial disease. Additionally, longitudinal follow-up of larger cohorts will be essential to fully understand the natural history and response to therapeutic interventions in this patient population.

## Supplementary Material

fcag095_Supplementary_Data

## Data Availability

All patient clinical and molecular data supporting the conclusions of this study are presented within the article, figures, and Supplementary material.

## References

[1] Balsa E, Marco R, Perales-Clemente E, et al NDUFA4 is a subunit of complex IV of the mammalian electron transport chain. Cell Metab. 2012;16(3):378–386. 22902835 10.1016/j.cmet.2012.07.015

[2] Carroll J, Fearnley IM, Skehel JM, Shannon RJ, Hirst J, Walker JE. Bovine complex I is a complex of 45 different subunits. J Biol Chem. 2006;281(43):32724–32727. 16950771 10.1074/jbc.M607135200

[3] Formosa LE, Muellner-Wong L, Reljic B, et al Dissecting the roles of mitochondrial Complex I intermediate assembly complex factors in the biogenesis of Complex I. Cell Rep. 2020;31(3):107541. 32320651 10.1016/j.celrep.2020.107541

[4] Nouws J, Nijtmans LG, Smeitink JA, Vogel RO. Assembly factors as a new class of disease genes for mitochondrial complex I deficiency: Cause, pathology and treatment options. Brain. 2012;135(Pt 1):12–22. 22036961 10.1093/brain/awr261

[5] Fernandez-Vizarra E, Zeviani M. Mitochondrial disorders of the OXPHOS system. FEBS Lett. 2021;595(8):1062–1106. 33159691 10.1002/1873-3468.13995

[6] Jackson TD, Crameri JJ, Muellner-Wong L, et al Sideroflexin 4 is a complex I assembly factor that interacts with the MCIA complex and is required for the assembly of the ND2 module. Proc Natl Acad Sci U S A. 2022;119(13):e2115566119. 35333655 10.1073/pnas.2115566119 PMC9060475

[7] Carroll J, He J, Ding S, Fearnley IM, Walker JE. TMEM70 and TMEM242 help to assemble the rotor ring of human ATP synthase and interact with assembly factors for complex I. Proc Natl Acad Sci U S A. 2021;118(13):e2100558118. 33753518 10.1073/pnas.2100558118 PMC8020751

[8] Oláhová M, Guerra RM, Collier JJ, et al RTN4IP1 is required for the final stages of mitochondrial complex I assembly and CoQ biosynthesis. EMBO J. 2025;44(19):5482–5508. 40859035 10.1038/s44318-025-00533-x PMC12489013

[9] Kirby DM, Crawford M, Cleary MA, Dahl HH, Dennett X, Thorburn DR. Respiratory chain complex I deficiency: An underdiagnosed energy generation disorder. Neurology. 1999;52(6):1255–1264. 10214753 10.1212/wnl.52.6.1255

[10] Ng YS, Thompson K, Loher D, et al Novel MT-ND gene variants causing adult-onset mitochondrial disease and isolated Complex I deficiency. Front Genet. 2020;11:24. 32158465 10.3389/fgene.2020.00024 PMC7052259

[11] Yu-Wai-Man P, Griffiths PG, Chinnery PF. Mitochondrial optic neuropathies—Disease mechanisms and therapeutic strategies. Prog Retin Eye Res. 2011;30(2):81–114. 21112411 10.1016/j.preteyeres.2010.11.002 PMC3081075

[12] Carelli V, La Morgia C, Yu-Wai-Man P. Mitochondrial optic neuropathies. Handb Clin Neurol. 2023;194:23–42. 36813316 10.1016/B978-0-12-821751-1.00010-5

[13] Chermakani P, Gowri P, Mahesh S, et al Exploring mito-nuclear genetic factors in Leber's hereditary optic neuropathy: Insights from comprehensive profiling of unique cases. EXCLI J. 2023;22:1077–1091. 38054206 10.17179/excli2023-6297 PMC10694345

[14] Darin N, Oldfors A, Moslemi AR, Holme E, Tulinius M. The incidence of mitochondrial encephalomyopathies in childhood: Clinical features and morphological, biochemical, and DNA abnormalities. Ann Neurol. 2001;49(3):377–383. 11261513

[15] Schlieben LD, Prokisch H. The dimensions of primary mitochondrial disorders. Front Cell Dev Biol. 2020;8:600079. 33324649 10.3389/fcell.2020.600079 PMC7726223

[16] Diodato D, Schiff M, Cohen BH, Bertini E, Rahman S. 258th ENMC international workshop Leigh syndrome spectrum: Genetic causes, natural history and preparing for clinical trials 25–27 March 2022, Hoofddorp, Amsterdam, The Netherlands. Neuromuscul Disord. 2023;33(8):700–709. 37541860 10.1016/j.nmd.2023.06.002

[17] McKelvie P, Infeld B, Marotta R, Chin J, Thorburn D, Collins S. Late-adult onset Leigh syndrome. J Clin Neurosci. 2012;19(2):195–202. 22273117 10.1016/j.jocn.2011.09.009

[18] Fassone E, Rahman S. Complex I deficiency: Clinical features, biochemistry and molecular genetics. J Med Genet. 2012;49(9):578–590. 22972949 10.1136/jmedgenet-2012-101159

[19] Torraco A, Nasca A, Verrigni D, et al Novel NDUFA12 variants are associated with isolated complex I defect and variable clinical manifestation. Hum Mutat. 2021;42(6):699–710. 33715266 10.1002/humu.24195

[20] Marsili L, Mantecon M, Arrondel C, et al Genome sequencing identifies RMND1 as a strong candidate gene for severe prenatal kidney failure mimicking renal tubular dysgenesis associated with hyporeninism. Pediatr Nephrol. 2025;40(9):2823–2828. 40366408 10.1007/s00467-025-06787-1

[21] Alston CL, Howard C, Oláhová M, et al A recurrent mitochondrial p.Trp22Arg NDUFB3 variant causes a distinctive facial appearance, short stature and a mild biochemical and clinical phenotype. J Med Genet. 2016;53(9):634–641. 27091925 10.1136/jmedgenet-2015-103576 PMC5013090

[22] Yépez VA, Gusic M, Kopajtich R, et al Clinical implementation of RNA sequencing for Mendelian disease diagnostics. Genome Med. 2022;14(1):38. 35379322 10.1186/s13073-022-01019-9 PMC8981716

[23] Taylor RW, Pyle A, Griffin H, et al Use of whole-exome sequencing to determine the genetic basis of multiple mitochondrial respiratory chain complex deficiencies. JAMA. 2014;312(1):68–77. 25058219 10.1001/jama.2014.7184 PMC6558267

[24] Asif M, Khayyat AIA, Alawbathani S, et al Biallelic loss-of-function variants of ZFTRAF1 cause neurodevelopmental disorder with microcephaly and hypotonia. Genet Med. 2024;26(7):101143. 38641995 10.1016/j.gim.2024.101143

[25] den Dunnen JT, Dalgleish R, Maglott DR, et al HGVS recommendations for the description of sequence variants: 2016 update. Hum Mutat. 2016;37(6):564–569. 26931183 10.1002/humu.22981

[26] Richards S, Aziz N, Bale S, et al Standards and guidelines for the interpretation of sequence variants: A joint consensus recommendation of the American College of Medical Genetics and Genomics and the Association for Molecular Pathology. Genet Med. 2015;17(5):405–424. 25741868 10.1038/gim.2015.30 PMC4544753

[27] García JJ, Ogilvie I, Robinson BH, Capaldi RA. Structure, functioning, and assembly of the ATP synthase in cells from patients with the T8993G mitochondrial DNA mutation. Comparison with the enzyme in Rho(0) cells completely lacking mtdna. J Biol Chem. 2000;275(15):11075–11081. 10753912 10.1074/jbc.275.15.11075

[28] Acín-Pérez R, Hernansanz-Agustín P, Enríquez JA. Analyzing electron transport chain supercomplexes. Methods Cell Biol. 2020;155:181–197. 32183958 10.1016/bs.mcb.2019.12.002

[29] Zerbetto E, Vergani L, Dabbeni-Sala F. Quantification of muscle mitochondrial oxidative phosphorylation enzymes via histochemical staining of blue native polyacrylamide gels. Electrophoresis. 1997;18(11):2059–2064. 9420170 10.1002/elps.1150181131

[30] Catania A, Ardissone A, Verrigni D, et al Compound heterozygous missense and deep intronic variants in NDUFAF6 unraveled by exome sequencing and mRNA analysis. J Hum Genet. 2018;63(5):563–568. 29531337 10.1038/s10038-018-0423-1 PMC6071912

[31] Hartmannová H, Piherová L, Tauchmannová K, et al Acadian variant of Fanconi syndrome is caused by mitochondrial respiratory chain complex I deficiency due to a non-coding mutation in complex I assembly factor NDUFAF6. Hum Mol Genet. 2016;25(18):4062–4079. 27466185 10.1093/hmg/ddw245

[32] Gedikbasi A, Toksoy G, Karaca M, et al Clinical and bi-genomic DNA findings of patients suspected to have mitochondrial diseases. Front Genet. 2023;14:1191159. 37377599 10.3389/fgene.2023.1191159 PMC10292751

[33] Hu C, Li X, Zhao L, et al Clinical and molecular characterization of pediatric mitochondrial disorders in south of China. Eur J Med Genet. 2020;63(8):103898. 32348839 10.1016/j.ejmg.2020.103898

[34] Kohda M, Tokuzawa Y, Kishita Y, et al A comprehensive genomic analysis reveals the genetic landscape of mitochondrial respiratory chain complex deficiencies. PLoS Genet. 2016;12(1):e1005679. 26741492 10.1371/journal.pgen.1005679 PMC4704781

[35] Fowler DM, Fields S. Deep mutational scanning: A new style of protein science. Nat Methods. 2014;11(8):801–807. 25075907 10.1038/nmeth.3027 PMC4410700

[36] Sung AY, Guerra RM, Steenberge LH, et al Systematic analysis of NDUFAF6 in complex I assembly and mitochondrial disease. Nat Metab. 2024;6(6):1128–1142. 38720117 10.1038/s42255-024-01039-2 PMC11395703

[37] Stroud DA, Surgenor EE, Formosa LE, et al Accessory subunits are integral for assembly and function of human mitochondrial complex I. Nature. 2016;538(7623):123–126. 27626371 10.1038/nature19754

[38] Guerrero-Castillo S, Baertling F, Kownatzki D, et al The assembly pathway of mitochondrial respiratory chain Complex I. Cell Metab. 2017;25(1):128–139. 27720676 10.1016/j.cmet.2016.09.002

[39] Johnstone T, Wang J, Ross D, et al Biallelic variants in two complex I genes cause abnormal splicing defects in probands with mild Leigh syndrome. Mol Genet Metab. 2020;131(1–2):98–106. 33097395 10.1016/j.ymgme.2020.09.008 PMC7749052

[40] McKenzie M, Tucker EJ, Compton AG, et al Mutations in the gene encoding C8orf38 block complex I assembly by inhibiting production of the mitochondria-encoded subunit ND1. J Mol Biol. 2011;414(3):413–426. 22019594 10.1016/j.jmb.2011.10.012

[41] Moreno-Lastres D, Fontanesi F, García-Consuegra I, et al Mitochondrial complex I plays an essential role in human respirasome assembly. Cell Metab. 2012;15(3):324–335. 22342700 10.1016/j.cmet.2012.01.015 PMC3318979

[42] Torraco A, Stehling O, Stümpfig C, et al ISCA1 mutation in a patient with infantile-onset leukodystrophy causes defects in mitochondrial [4Fe-4S] proteins. Hum Mol Genet. 2018;27(15):2739–2754. 29767723 10.1093/hmg/ddy183

[43] D'Angelo L, Astro E, De Luise M, et al NDUFS3 depletion permits complex I maturation and reveals TMEM126A/OPA7 as an assembly factor binding the ND4-module intermediate. Cell Rep. 2021;35(3):109002. 33882309 10.1016/j.celrep.2021.109002 PMC8076766

[44] Imai-Okazaki A, Kishita Y, Kohda M, et al Cardiomyopathy in children with mitochondrial disease: Prognosis and genetic background. Int J Cardiol. 2019;279:115–121. 30642647 10.1016/j.ijcard.2019.01.017

[45] Ogawa E, Fushimi T, Ogawa-Tominaga M, et al Mortality of Japanese patients with Leigh syndrome: Effects of age at onset and genetic diagnosis. J Inherit Metab Dis. 2020;43(4):819–826. 31967322 10.1002/jimd.12218 PMC7383885

