# Supplementary material for: Neurological manifestations and genotype–phenotype correlations in NDUFAF6-associated mitochondrial disease
Source: Brain Commun. 2026 Mar 18;8(2):fcag095. doi: 10.1093/braincomms/fcag095 (PMC13036829; doi:10.1093/braincomms/fcag095)
Supplement: fcag095_Supplementary_Data [file fcag095_supplementary_data.pdf]

SUPPLEMENTARY MATERIAL

Table of contents

Supplementary Results

Supplementary literature. References provided for Supplementary Tables 2 and 3.

Supplementary Table

- Supplementary Table 1. Clinical, genetic and MRI features.
- Supplementary Table 2. Genetic findings.
- Supplementary Table 3. Clinical, genetic and MRI features in published *NDUFAF6* mutated patients.

Supplementary Figures

- Supplementary Fig. 1. Neuroimaging study of P1.
- Supplementary Fig. 2. mRNA evaluation in P16.
- Supplementary Fig. 3. BN-PAGE of the assembly status of CI.

Supplementary Files

- Supplementary files 1-5. Uncropped blots.

Supplementary Results

Patients' description

Family 1 (P1, P2).

P1 is a 20-year-old male patient and the first child of healthy unrelated Italian parents. He was born at the 40th week of gestation by elective caesarean section after uneventful pregnancy. His medical history was characterized by failure to thrive and poor growth up to 8 months of age, when he was hospitalized at Bambino Gesu' Children's Hospital for diagnostic assessment. Clinical examination documented hyposomia (weight and height both <3° percentile) and mild hepatomegaly. During admission, he presented an acute episode of profuse sweating and lethargy. Severe hypoglycaemia (0.6 mmol/L, n.v. >2.6) and metabolic acidosis were detected. A diagnostic 12 h fasting test was then performed. The test was interrupted at the 9th hour for the evidence of non ketotic hypoglycemia (2.3 mmol/L), unresponsive to glucagon administration, with metabolic acidosis (pH 7.29; base excess -13 mmol/L, HCO3- 11.4 mEq/L), hypertransaminasemia (GOT 438 UI/l, n.v. <40; GPT 357 UI/l n.v. <40), hyperlactacidemia (5.9 6 mmol/L n.v.<2.2). At the end of the test, plasma insulin was < 2 µIU/mL (n.v.: 6- 27), cortisol 18.93 µg/dL (n.v.: 4-22), growth hormone (GH) 3.33ng/mL (n.v.: < 10). Blood ammonia, thyroid function, uric acid, NEFA and CPK levels were in the normal range. Urine organic acids analysis revealed markedly increased excretion of 3-OH-butyric and 3-OH-sebacic acids and medium increase of adipic, 3-OH dodecanedioic, fumaric, malic, succinic, lactic and 2 Ethyl-3OH propionic acids. Blood acylcarnitine profile showed a reduction in C2 and free carnitine levels. An abdominal ultrasonography documented marked hepatomegaly with inhomogeneous and *hyperechoic* abnormalities in liver and gall-bladder stones. The patient was discharged at home with a starch-enriched diet. After few months he developed a renal tubulopathy with proteinuria, aminoaciduria, hyperuricuria, tubular acidosis and increased β2 microglobulin. Suspecting a mitochondrial disease affecting the liver and the kidney, muscle, liver and skin biopsies were performed at 11 months. Histological and histochemical morphology of muscle biopsy showed no relevant abnormalities. Spectrophotometric determination of the activities of respiratory chain

complexes in muscle documented a 75% and a 51% decrease of complex I and complex IV activities, respectively. ATP synthesis analysis on mitochondria isolated from skin biopsy documented a reduction of complex V activity with all the substrates used (Succinate: -10%; Malate: -55%, Pyruvate/Malate: -53%). Liver histology highlighted a preserved architecture with microvesicular steatosis and hyperplasia of Ito cells. Hepatocytes had an oncocytic appearance due to a marked increase of the number of mitochondria, sometimes with mega-mitochondria. Electron microscopy confirmed accumulation of mitochondria, some of which had dysregulated cristae and a variable aspect of the matrix (granular or hyperdense). Moreover, an accumulation of lipids drops was observed. A brain MRI with spectroscopy performed at 13 months gave normal results (**Supplementary Fig. 1 A-A'**). He was then supplemented with Coq10 (20mg/kg, Carnitine (100 mg/kg) and alkalizing therapy with K citrate and Na bicarbonate. At 26 months of life he was admitted again for hypoglycemia, severe acidosis and acute appearance of right upper limb hemiparesis during fever. To prevent the possibility of hypoglycemia, the patient was treated with nocturnal enteral nutrition after positioning a percutaneous endoscopic gastrostomy, with clear clinical improvement, good blood glucose control, and no further episodes of acute metabolic decompensations. The hemiparesis resolved with physical therapy and he reached normal gross motor and cognitive functions. A new brain MRI at 24 months diagnosed a marked alteration of bi-hemispheric white matter with pseudo cystic appearance and involvement of the corpus callosum (**Supplementary Fig. 1 B-B'**). Serial MRI studies documented a stability of brain lesions in the first 7 years (**Supplementary Fig. 1 C-C', D-D' and E**). However, he continued to present poor growth, metabolic acidosis, tubulopathy and developed renal glomerular damage, compatible with Fanconi syndrome, that progressed up to chronic kidney failure. Additional clinical signs that became evident in the last 2 years of follow up were: renal hypertension, with slight ectasia of the ascending aorta and minimal mitral valve insufficiency, and right eye retinal macular dystrophy at Optical Coherence Tomography (OCT). Electroretinogram (ERG) highlighted that amplitude responses were within normal limits in both eyes but a with a difference in interocular amplitude (Left eye > Right eye).

Last neurological examination at 17 years of age disclosed no abnormalities. Last brain MRI performed at 14 years documented some increase in the extension of the white matter lesions particularly evident at T2/FLAIR with hyperintensity at level of subcortical and deep white matter in frontal and supra/paratrigonal regions bilaterally, and malacic evolution of the cystic areas, more evident in correspondence of the corpus callosum (**Supplementary Fig. 1 F-F', G, and H-H'**). However, there was no involvement of basal ganglia and brain stem. The spectroscopic study documented a slight increase in the NAA peak in absence of a lactate peak.

P2 is the younger brother of P1. He was born at 35weeks+6 by elective caesarean after a pregnancy complicated by maternal hypothyroidism and hyperprolactinemia. His birth parameters were in the normal range for age and Apgar was 8 at 1' and 9 at 5'. After a few hours, the child manifested acute respiratory distress and worsening of general conditions with poor reactivity, pallor, hypothermia, gastric bleeding. He was then ventilated, and transferred to the ICU of "Bambino Gesù" Children's Hospital. During the admission he underwent ventilation with nasal CPAP and oxygen therapy for 4 days. Gastric bleeding was resolved with physiological solution and Maalox and intravenous therapy with ranitidine. Routine laboratory tests documented: hyperbilirubinemia (maximum total bilirubin 13.5 mg- n.v. 0.25-1 mg/dl- with direct bilirubin 0.89 mg/dl – n.v. 0.8 -0.25), for which he underwent phototherapy for 3 days: non–haemolytic anaemia (Hb 10.6 g/dl, n.v. 13-16), treated with transfusions of concentrated red blood cells; mild pre-prandial asymptomatic hypoglycaemia on one occasion. A brain ultrasound gave normal, results, whereas ultrasound abdominal scan documented mild hepatomegaly. Standard audiometric examination and eye examination were normal. In consideration of the positive family history, metabolic exams were requested, which documented hyperlactacidemia (3.5 - 7.5 mmol/L) metabolic acidosis, slight increase in plasma alanine (466 µmol/l, n.v. 150-400), glutamine (974 µmol/l, n.v.200-800), glycine 427 µmol/l, n.v. 20-340) and creatine (106 µmol/l, n.v. 16-93) levels and presence of lactic and pyruvic acid in the urine. Blood ammonia and acylcarnitine profile were unremarkable. He was discharged at home at 18 days in good condition. He continued to experience hyporegenerative anaemia, tendency to hypoglycaemia, and hyperlactacidemia, which

required supplementation with folic acid and iron, a starch-enriched diet, and antioxidants therapy with Coq10 (20 mg/kg) and riboflavin (20 mg/kg). He was admitted again at 1 and 4 months of age for repeated episodes of acute respiratory distress, hypoglycaemia and severe metabolic acidosis during upper airway viral infections which required during both events rhino-tracheal intubation and mechanic ventilation. During a new admission at 6 months because of metabolic acidosis (pH 7.17; base excess -18 mmol/L, HCO_3^- 10.5 mEq/L), the child presented a rapid worsening of his clinical condition. Despite respiratory assistance, he developed a mixed metabolic and respiratory acidosis unresponsive to $NAHCO_3$ infusion, continuous veno-venous hemofiltration and mechanic ventilation, with progressive deterioration of his ventilation capacity and cardio-circulatory arrest. A skin-muscle biopsy was performed before his death. Histological and histochemical morphology of muscle biopsy showed no relevant abnormalities. Spectrophotometric determination of the activities of respiratory chain complexes in muscle showed Complex I and II activities at the lower limits of the normal ranges. ATP synthesis analysis on mitochondria isolated from skin biopsy documented a reduction of Complex V activity with all the substrates used (Succinate: -20%; Malate: -74%, Pyruvate/Malate: -65%).

Family 2 (P3, P4).

The two siblings were born at term from non-consanguineous Italian parents, with an age difference of 5 years. For both siblings, the postnatal period and early childhood were characterized by regular growth, in the absence of infectious events, feeding and sleep problems, or abnormal psychomotor development. P3 showed birth weight of 3990 gr (+1.21 SD), length of 53 cm (+0.61 SD), occipital frontal circumference (OFC) was 35 cm (+0.04 SD) and Apgar was 6 and 8 at the first and the fifth minute, respectively, because of fetal distress. However, the vital signs normalized and brain ultrasound did not reveal any significant anomalies. He showed an uneventfully psychomotor development up to 30 months of age when he manifested frequent falls that at 3 years led him to orthopedic evaluation. At 4 years brain magnetic resonance imaging (MRI) showed a bilateral involvement of caudate and putamen nuclei with T2 hyper-intensity also affecting left cerebellar

dentate nucleus together with areas of bilateral necrosis of the lenticular nuclei and monolateral necrosis of the right caudate nucleus with rounded shape. At the age of 5 years, he started to show deterioration in gross motor and language difficulties that progressively exacerbated, with dystonic movements and decreased fine motor abilities. At the time of the first examination in the genetics outpatient clinic he was 5 years and 6 months old and showed a dystonic spastic clinical picture in the absence of dysmorphism. His karyotype was normal, as the CGH array. At the age of 5y a skin and muscle biopsy were performed. Histological and histochemical morphology of muscle biopsy showed no relevant abnormalities. ATP synthesis analysis on mitochondria isolated from skin biopsy documented a reduction of Complex V activity with all the substrates used (Succinate: -27%; Malate: -60%, Pyruvate/Malate: -58%).

P4 had normal biometrics at birth, with Apgar 9 at 1 and 5 minutes due to uneventful labor. His regression in gross motor and speech began at 4 years and 6 months. He showed progressive dystonic movements and decreased fine motor skills, similarly to his brother, confirming that he was affected by the same disorder. A brain MRI disclosed bilateral striatal necrosis.

The clinical course of the two brothers showed slow disease progression during a 10-year long-term follow-up in rehabilitation. The two brothers are seated in wheelchairs, unable to stand for more than a few seconds with support. They have markedly decreased fine hand motor skills due to dystonic movements. Verbal comprehension remains preserved, but verbal production is characterized by severe dysarthria.

Family 3 (P5).

P5 was born at term after a normal-running pregnancy, second son of two siblings, from non-consanguineous parents. No problems at birth were reported. Early motor development was normal: he gained autonomous ambulation at 16 months of age. He presented speech delay. At the age of 2, the parents noted loss of balance with frequent falls. Over time, the child had lost the ability to walk. He was then admitted to the Neurology Unit and at the examination showed normal ocular motility, dysarthria, axial hypotonia, spastic-dystonic tetraparesis with bilateral Babinski sign; he was able to

maintain the sitting position and to crawl but not to walk. Metabolic investigations (plasma aminoacids, urinary organic acids and acilcarnitine) were in the normal range. Brain MRI showed bilateral and symmetric hyperintensity in T2-weighted images in correspondence of the putamen, pallid nuclei, cerebral peduncles, mesencephalic and pontine tegmentum, cerebellar dentate nuclei, right thalamus and peri-aqueductal gray matter with a peak lactate at spectroscopy; electroretinogram, visual evoked and brainstem potentials were normal. Histochemical of muscle biopsy showed diffuse reduction of NADH. ATP synthesis analysis on mitochondria isolated from skin biopsy displayed a normal Complex V activity. He started treatment with CoQ10 and riboflavin. In the following months, the child started physiotherapy and showed a progressive motor improvement regaining the ability to walk. The clinical picture remained fairly stable until the age of 4. He repeated a brain MRI at the age of 4 that showed reduction of the volume and swelling appearance of the putamen and caudate nucleus bilaterally, with reduction of the areas of restriction of diffusivity in correspondence with the T2/FLAIR hyperintense areas at that site, in relation to the evolution of underlying pathology; lesions of the dentate nuclei where almost no longer appreciable in comparison with the previous MRI exam. Emogas analysis revealed mild metabolic acidosis and a treatment with sodium bicarbonate was started; Holter-ECG and echocardiogram were normal. His motor picture continued to improve until the age of 4 years and a half, when he showed again frequent falls and more loss of balance and of autonomous ambulation. He repeated metabolic workout and neurophysiological assessment (VEP, ERG and BAEPs) that gave normal results and a brain MRI that showed no differences compared to the previous exam.

Family 4 (P6).

P6, the only child of non-consanguineous healthy parents of French origin, was born after a normal pregnancy and eutocic term delivery with normal birth parameters (BW: 2.9 Kg, BH:52cm, BHC:35cm, Apgar 9 and 10). Psychomotor development was normal till the age of 2 years when he started to present gait instability. At 2,2 years, neurological examination showed left hemiparesis and dystonia. The child lost the ability to walk and to control his head at 3 years. Metabolic work-up

showed increased plasma lactate (2.3-4.6 mmol/L, normal <2) and lactate/pyruvate molar ratios (18, normal 6-14). Plasma amino acids, liver enzymes (ASAT, ALAT) and urinary organic acids were normal. Muscle biopsy showed a normal histology. Respiratory chain essay disclosed a complex I deficiency in muscle and fibroblasts. ECG, heart ultrasonography, EEG, electromyography, fundus oculi and ophthalmic examination were normal. Brain MRI at 2.3 years of age demonstrated bilateral and symmetric T2 hyperintensity of basal ganglia characteristic of Leigh disease. The child died at 12 years due to respiratory distress in a context of inhalation pneumonia.

Family 5 (P7, P8, P9).

P7 is the first child of non-consanguineous healthy parents of French origin from Saudi Arabia. He is born after a normal pregnancy and eutocic term delivery with normal birth parameters. Early psychomotor development was normal. At 11 months he started to walk and pronounce his first words. At the age of 3 years, the parents noted gait instability with frequent falls. He started to show a slow motor regression with dystonic movements and decreased fine motor abilities. The child lost the ability to walk at 6 years. He also presented a progressive dysarthria. Neurological examination at 12 years showed dysarthria, axial hypotonia, spastic-dystonic tetraparesis with bilateral Babinski sign; he was not able to sit.

P8 is the sister of P7 and P9. She was born at term after a regular pregnancy. No problems at birth were reported. Early psychomotor development was reported as normal. At the age of 5 years, the parents noted gait instability with frequent falls and dysarthria. The child lost the ability to walk at 9 years. Neurological examination showed dysarthria, axial hypotonia, and spastic-dystonic tetraparesis with bilateral Babinski sign.

P9 is the youngest sister of P7 and P8. She is born after a normal pregnancy and eutocic term delivery with normal birth parameters. Early psychomotor development was mildly delayed (walked at 15 m, had language difficulties). At the age of 5 years, the parents noted gait instability with frequent falls and dysarthria. The child lost the ability to walk at 6 years. Neurological examination showed dysarthria, axial hypotonia, and spastic-dystonic tetraparesis with bilateral Babinski sign.

Metabolic investigations (plasma aminoacids, urinary organic acids and acilcarnitine) were in the normal range. Brain MRI (for P7 and P8) showed bilateral and symmetric hyperintensity in T2-weighted images of the putamina and caudate nuclei. Spectroscopy was performed for P7 and showed a lactate peak. Respiratory chain analysis showed a deficit of complex 1 in muscle sample of P9.

Family 6 (P10).

P10 is the fifth child of healthy not-consanguineous parents of French origin. She was born at term after a regular pregnancy by eutocic delivery. She was hospitalized at 2 months for lack of reactivity. Clinical examination showed global hypotonia and lack of contact. Laboratory investigations revealed a lactic acidosis. She deceased at the age of 3 months. Respiratory chain essay disclosed a complex I deficiency in muscle.

Family 7 (P11).

P11 is the second child born to non-consanguineous healthy parents of French origin. She was born at term after a regular pregnancy and delivery. Apgar was 10/10. Birth growth parameters were below the 5° percentile (BW 2360gr, BH 44cm, BHC 33cm). She was hospitalized at 5 weeks for lack of reactivity. Clinical examination showed axial hypotonia, peripheral hypertonia, and lack of contact. She also showed clonic legs movements. Multifocal epileptic seizures were recorded on EEG. Laboratory investigations revealed a lactic acidosis (blood pH 7.2, normal 7.4; plasma lactate concentration up to 10.8 mmol/l, normal<2.20 mmol/l) and a high lactate/pyruvate ratio. Lactate concentration in cerebrospinal fluid was increased (8 mmol/L, normal < 2). She was transferred to the ICU and treated by i.v. phenytoin. Brain MRI (5 weeks) showed bilateral basal ganglia T2 hyperintensity suggestive of Leigh syndrome and cortical hyperintensity on diffusion weighted images. Respiratory chain essay disclosed a severe complex I deficiency in muscle and fibroblasts. Blue native page showed qualitative and quantitative complex I assembly abnormalities on fibroblasts. The child deceased at 6 weeks.

Family 8 (P12).

P12 is a 14-year-old girl born to a consanguineous family of Moroccan descent (parents are first

cousins). The couple had a history of 1 miscarriage (after 2 months of gestation). At 3 years, parents observed a psychomotor regression: she presented gait unsteadiness progressing to a loss of walking, sit and head control. She also lost verbal language. She started to present vomiting episodes with dehydration. Cerebral MRI performed at the age 6 years old revealed cortical atrophy with periventricular white matter abnormalities while laboratory testing showed abnormal organics acids (increase in 2-hydroxyisobutyric acid and 3-hydroxypropionic acid without methyl citric acid). At followup (10 years), she had a severe global hypotonia with dystonic movements of legs and arms, and she could not control her head. She preserved the contact and she can pronounce a few words. Metabolic work-up showed increased plasma lactate (3-4.1 mmol/L, normal <2). Whereas, plasma amino acids, liver enzymes (ASAT, ALAT) and urinary organic acids were normal. A second brain MRI performed at 10 years showed symmetrical basal ganglia T2 hyperintensity evocating Leigh syndrome. Whole mitochondrial DNA sequencing was normal. She had 3 older siblings, 2 brothers who passed away early in life (aged 2-5 years old) presenting with a similar condition, and a 19-year-old healthy brother.

Family 9 (P13).

P13 is the first child of healthy unrelated parents of Moroccan origin, born at term after uneventful pregnancy and eutocic delivery. First psychomotor development was reported as regular by the parents. From the age of 3 years, he started to present gait difficulties and a left leg dystonia was observed at neurological examination. He started to show a slow regression in gross motor and language difficulties that progressively exacerbated, with dystonic movements and decreased fine motor abilities. At the time of the last examination in genetics outpatient clinic he was 10 years and showed a dystonic spastic clinical, could walk with help for a short distance. Verbal language comprehension was preserved, but verbal production was characterized by severe dysarthria. At 3, 6- and 10-years brain MRI showed bilateral basal ganglia T2-hyperintensity involving putamen, lenticular and caudate nuclei. Metabolic work up consistently showed elevated plasma lactate levels and lactate/pyruvate molar ratios (lactate: 3 mM, normal values below 2.4 mM, L/P ratio: 20-22,

normal values 10-12). Respiratory chain essay disclosed a complex I deficiency in fibroblasts.

Family 10 (P14, P15).

P14 was born at term after a normal pregnancy by caesarean section. Both parents are second cousins. He was born in good condition without admission to SCBU. He reached his early milestones in his first few months of life, but parents had concerns regarding his development by 9 months of age, when he was unable to sit unsupported. At 3 years of age, he had difficulty standing independently. Swallowing deteriorated at this time and he had excessive drooling when eating. He subsequently required percutaneous gastrostomy feeding. Paediatric neurology team requested skeletal muscle biopsy which revealed an isolated complex I deficiency. A cranial MRI showed bilateral basal ganglia and brainstem involvement. Plasma and CSF lactate were noted to be elevated. He continued to have central hypotonia, but his peripheral tone gradually increased. He also had severe dysphonia and required an electronic communication aid. His medications include Diazepam, Glycopyrronium, Movicol, Coenzyme Q10, riboflavin and Vitamin D. He had regular health surveillance with the paediatric and adult teams. He is 21 years old at the moment. He is living in supported accommodation but has agoraphobia and anxiety issues following the unexpected death of his sibling (P15).

P15 is the sibling of patient 14 and was born at term after a normal pregnancy by caesarean section for previous C-section. He was born in good condition with birth weight of 2.36kg. He met his early milestones and gained weight. He sat independently at eight months and stood at thirteen months. He had abnormal left foot posture at 18 months of life. His gross motor delay became more apparent at 14 months of age. He was diagnosed with the same condition as his brother at this age. He had axial hypotonia with dystonia and subsequently spasticity in his limbs. His language comprehension was appropriate for his age, but at around 22 months he developed speech and swallowing difficulties, coughing and choking on liquids and was subsequently diagnosed with dysphonia and dysphagia. His MRI showed bilateral, symmetrical, abnormal low T1 and high T2 signal change within the putamina, parietal white matter and dorsal pons. He was started on medications including Trihexiphenidyl, Diazepam, CoQ10, riboflavin, Co-enzyme Q10 and Movicol. His ECG and echocardiogram were

normal throughout his annual surveillance. He was a wheelchair user with limited ability to move on his own. He received nutrition via percutaneous gastrostomy feeding. This boy died at the age of 9 years old. He was found to be unresponsive and not breathing. Out of hospital CPR was commenced but was unsuccessful. The post-mortem concluded that it is likely that he died from natural causes with arrhythmia or first seizure as the most likely mechanism of death.

Family 11 (P16).

Limited details as reported in Supplementary Table 1

Family 12 (P17 and P18).

P17 was born at term in good condition but developed severe lactic acidosis at 18 hours of age. This lactic acidosis was not correctable despite interventions from the neonatal team. Her muscle biopsy showed isolated Complex 1 deficiency. She did not have any MRI of her brain. She died in the neonatal intensive care unit at 9 weeks of age from lactic acidosis.

P18, for this patient no medical history available.

Family 13 (P19, P20, P21).

P19 was distantly related to patient 17. She was born in good condition and was discharged home. She had viral bronchiolitis at 3 months of life. She became increasingly drowsy and she then had sudden deterioration. She was resuscitated and artificially ventilated in a paediatric intensive care unit, where blood results showed a significant metabolic acidosis. A subsequent skeletal muscle biopsy showed isolated Complex I deficiency. She died at 3 months of life from bronchiolitis and metabolic acidosis.

P20 an P21 are the deceased aunt and uncle of patient 19, no medical history is available for both.

Family 14 (P22).

P22 was born at term after a normal pregnancy with normal birth parameters (BW: 3.1 Kg, BH:53cm, BHC:35.2cm, Apgar 7/8) from first degree cousin marriage. He was hospitalized at 3 months for slow activity. Clinical examination showed axial hypotonia, slight peripheral hypertonia, and limited contact. During hospitalization convulsive movements were observed. EEG showed multifocal spikes

and background slowing. Laboratory investigations revealed a lactic acidosis (blood pH 7.1, normal 7.4; plasma lactate concentration: 11 mmol/l, normal<2.20 mmol/l) and a high lactate/pyruvate ratio. Lactate concentration in cerebrospinal fluid was increased (7.2 mmol/L, normal < 2). Liver and renal function analyses were normal, blood CK was 171 IU. Brain MRI (3 months) showed bilateral basal ganglia T2 hyperintensity suggestive of Leigh syndrome and cortical hyperintensity on diffusion weighted images. Dichloroacetate (DCA) and biotin, thiamin, creatin and coenzyme Q treatment was started. A modified ketogenic diet (KD, 3:1) with MCT (medium chain triglyceride) was started at age of 4 months. Afterwards starting KD, he showed improvement in neurodevelopmental milestones. At 8 months of age, he had completely head control, sitting with support and eye contact. He was hospitalized 2 times for hyperlactatemia (5.1 and 4.9 mmol/l) during infections (bronchiolitis and pneumonia). The child deceased at 9 months when he was in intensive care unit because of pneumonia.

Family 15 (P23).

P23 was third child of second-degree consanguineous parents, with family history of multiple sudden infant death story. She was born at 38 gestational weeks by emergency cesarean section due to growth arrest. Birth weight was 2,085 g (–2.61 standard deviation [SD]), height 46 cm (–1.76 SD), and head circumference 31 cm (–2.49 SD). She presented severe lactic acidosis and antiepileptic resistant convulsions at the 2nd day of life. Because blood lactate concentration reached to 18 mmol/l (normal values: <2.2), continuous venovenous hemodialysis (CVVHD) was started. Lactate concentration was normalized with CVVDH. Her MRI showed deep cerebral and cerebellar white matter and basal ganglia hyperintensity consistent with Leigh syndrome. Mitochondrial cocktail (Biotin, thiamin, coenzyme Q, lipoic acid, vitamin E) was started. At 6 m of age she had peripheric hypertonicity, slightly developmental delay with head control, rolling slowly but not sitting with support. Ophthalmologic examination showed optic atrophy. Metabolic work-up showed proximal tubular acidosis. After leaving the hospital (6/12 m), we could not reach the family and they did not bring the child for follow-up. She was referred to our hospital at 4 years old with severe lactic acidosis. When

we accepted her, her body weight was 8 kg, height 110 cm (<-2 SDS, <-2SDS). Because she was unconscious we could not make neurological examination in a proper way but the family told that she did not have head control and eye contact. Physical examination revealed dysmorphic features as triangular face, deep set nasal bridge, thin chin, short neck, severe cachexia and extremity contractures. The child deceased at 4 y of age during ICU hospitalization.

Family 16 (P24 and P25).

P24 was delivered at 32 weeks gestational age by C-section to second degree consanguineous parents of primarily Syrian descent. No family history of neuromuscular disorders. Mother noted vigorous in utero movements similar to their older healthy brothers. She had hyperbilirubinemia requiring phototherapy during her hospitalization because of prematurity and feeding difficulty. Her blood gas and laboratory analyses were normal then. First concerns were raised with movement delay (sitting with support was at 11 months with poor eye contact). At 15 months of age she developed multiple episodes of projectile vomiting, metabolic acidosis, and elevated levels of lactate (> 7 mmol/L, normal < 2.1), plasma alanine (> 950 μmol/L, normal < 495). Urine organic acids showed increased excretion of lactate, pyruvate and the citric acid cycle intermediate fumarate (>212 mg/gm creatinine; normal < 40); a pattern suggestive of an underlying oxidative phosphorylation disorder. Physical exam showed severe hypotonia but appendicular structures were hypertonic, lower extremities more involved than upper extremities also with delay in gross motor, fine motor, language. Also, we observed a left eye gaze preference with occasional random nystagmoid movements. Pupils were sluggishly reactive to light, but there was no blink to bright light or confrontation. Optic nerves were pale bilaterally; all other cranial nerves were grossly normal. Her EEG demonstrated generalised epileptic activity. Based on the EEG findings, started levetiracetam, and was subsequently placed on a ketogenic diet which, after 2 months, was discontinued due to persistent vomiting. MRI images showed abnormal T2 signal hyperintensity diffusely involving the periventricular white matter, external capsule, corpus callosum, and thalami bilaterally which were enlarged, edematous and swollen. There were also areas of associated restricted diffusion. Brain MRS in the centrum semiovale

showed a large lactate peak and reduced N-acetyl-aspartate (NAA) peak. Echocardiography showed left ventricular hypertrophy. Her muscle biopsy showed increasing of lipid droplets and abnormal fiber configuration. She passed away at 18 m of age because of covid-19 pneumonia.

P25 is the 3.5-year-old full male sibling of P24 who presented with a similar albeit milder phenotype, with febrile seizures at 26 months. He was born at 39 weeks gestation, by spontaneous vaginal delivery, weighing 3,85 kg. During the pregnancy, a prenatal diagnosis was made since the female sibling's history. The family did not accept the termination. He started on oral riboflavin, biotin, thiamine, coenzyme Q10, L-carnitine. Cranial MRI showed signal abnormalities involving deep white matter, optical nerve and cervical spine. His plasma and urine aminoacids showed mild proximal renal tubular acidosis (urine organic acids, plasma lactate levels, liver and renal enzymes were normal). Electromyography/nerve conduction studies were normal. At last physical examination his growth was normal to age, had head control, ataxic gait and was able to speak 20 words. Ophthalmological and audiological exams as well as ECHO were normal.

Family 17 (P26).

P26 is a 7-year-old female presented with early onset developmental delay and absence seizures at 3.5 years. She was born at 33 weeks gestation, by spontaneous vaginal delivery, weighing 1.24 kg. She presented during the first months of life with failure to thrive and mild neuromotor developmental delay: she started smiling at 2 months, head control was attained at the age of 6 months, she never acquired the ability to sit without support nor to roll. At 12 months he started babbling. She was first referred to pediatric metabolism department at the age of 5 years, when her parents noted that she had become somnolent and irritable. Neurological examination disclosed severe neuromotor delay and reduction of spontaneous movements. Laboratory studies revealed a slight increase of transaminases (aspartate transaminase [AST] 127 IU/L, alanine transaminase [ALT] 173 IU/L); liver ultrasounds and other liver function test were normal. During the following days her parents observed a rapid deterioration, hence she was hospitalized for diagnostic workup. At admission, she presented with marked irritability, truncal hypotonia with mild pyramidal signs at four limbs, and mild swallowing

difficulties. She had lost head control and was no longer able to smile or babble. Routine blood tests confirmed an increase in liver function tests (AST 142 IU/L, ALT 177 IU/L; gamma-glutamyl transferase was normal), but normal creatinine kinase, glucose, and ammonia levels; also, lactate level was increased (7.36 mmol/L). Electroencephalogram (EEG), electrocardiogram, and echocardiogram were all normal. Brain MRI showed a signal abnormality on corpus striatum, nucleus caudatus, globus pallidus, putamen, medial thalamic nucleus and brain stem, consistent with Leigh Syndrome. An abnormally high peak of lactate was visible on MRS within the abnormal white matter. Multiple areas of diffusion restriction were seen within the abnormal cerebral white matter, but not in the cerebellar abnormal areas. Areas of contrast enhancement mainly in the rims of the cystic areas in the centrum semiovale were also present. Muscle histological examination was normal. She is 7.5 years old at the moment, had head control, is able to sit with support and is under DCA, riboflavin, biotin, thiamine, coenzyme Q10, L-carnitine.

Family 18 (P27).

P27 is a 4-year-old boy with a global developmental delay who primarily had motor skills issues. His unrelated Iraqi parents separated when he was two years old. He had a near-term (36 – week gestational age) caesarean section birth after a normal pregnancy and was admitted to the neonatal intensive care unit after experiencing dyspnea and receiving oxygen for two days. At 20 months of age he began to walk, but he claimed to fall a lot. He was referred to the pediatric neurology clinic on November 2023 for examination and evaluation. The boy was socially responsive and alert. His OFC was 50 cm. He had a persistently open mouth due to oral dystonia and a weak cough. When seated, axial hypotonia and weakness cause bending back. When standing and walking, he had asymmetrical gait with dystonic posturing of the right arm and leg. Because of his decreased muscle mass, he showed thin body built. He had dysarthria, drooling saliva from a mouth that was always open (dystonia), hypertonia of the upper and lower limbs, decreased DTRs in the upper limbs, and increased DTRs in the lower limbs and flexor planters. His speech and motor skills had deteriorated more, especially in the last two months. His gait deteriorated, especially on the right side, which

showed dystonic hand and arm posture. His EMG/NCS revealed myopathic changes and brain MRI showed abnormal bilateral hyperintense lesions (T2WI/FLAIR) involving putamen and globi pallidi. WES revealed compound heterozygous variants in *NDUFAF6* gene. He started treatment with COQ10, L-carnitin, riboflavin and thiamin. Trihexyphenidyl together with gabapentin were added as symptomatic treatment of dystonia.

Supplementary literature. References provided for Supplementary Tables 2 and 3.

Catania A, Ardissone A, Verrigni D, *et al*. Compound heterozygous missense and deep intronic variants in NDUFAF6 unraveled by exome sequencing and mRNA analysis. *J Hum Genet*. 2018;63(5):563-568.

Hartmannová H, Piherová L, Tauchmannová K, *et al*. Acadian variant of Fanconi syndrome is caused by mitochondrial respiratory chain complex I deficiency due to a non-coding mutation in complex I assembly factor NDUFAF6. *Hum Mol Genet*. 2016;25(18):4062-4079

Gedikbasi A, Toksoy G, Karaca M, *et al*. Clinical and bi-genomic DNA findings of patients suspected to have mitochondrial diseases. *Front Genet*. 2023;14:1191159

Hu C, Li X, Zhao L, *et al*. Clinical and molecular characterization of pediatric mitochondrial disorders in south of China. *Eur J Med Genet*. 2020;63(8):103898.

Kohda M, Tokuzawa Y, Kishita Y, *et al*. A Comprehensive Genomic Analysis Reveals the Genetic Landscape of Mitochondrial Respiratory Chain Complex Deficiencies. *PLoS Genet*. 2016;12(1):e1005679.

Zhou Y, Zeng X, Zhang L, *et al*. Biallelic variants in the *NDUFAF6* cause mitochondrial respiratory complex assembly defects associated with Leigh syndrome in probands. *Mol Genet Metab Rep*. 2024 Dec 5; 41:101168.

Kim J, Lee J, Jang DH. *NDUFAF6*-Related Leigh Syndrome Caused by Rare Pathogenic Variants: A Case Report and the Focused Review of Literature. *Front Pediatr*. 2022 May 18;10:812408.

Johnstone T, Wang J, Ross D, *et al.* Biallelic variants in two complex I genes cause abnormal splicing defects in probands with mild Leigh syndrome. *Mol Genet Metab*. 2020;131(1-2):98-106.

Baide-Mairena H, Gaudó P, Marti-Sánchez L, *et al.* Mutations in the mitochondrial complex I assembly factor NDUFAF6 cause isolated bilateral striatal necrosis and progressive dystonia in childhood. *Mol Genet Metab*. 2019; 126(3):250-258.

Pagliarini DJ, Calvo SE, Chang B, *et al.* A mitochondrial protein compendium elucidates complex I disease biology. *Cell.* 2008 Jul 11;134(1):112-23.

Martikainen MH, Ng YS, Gorman GS, *et al*. Clinical, Genetic, and Radiological Features of Extrapyramidal Movement Disorders in Mitochondrial Disease. *JAMA Neurol.* 2016 Jun 1;73(6):668-74.

Supplementary Table 1 Clinical, genetic and MRI features in our cohort of *NDUFAF6* mutated patients

Family	Patient	cDNA and protein change	Sex	Pregnancy and Birth	Consanguinity	Age at onset/symptoms	Neurological picture	Clinical course/age at last follow up	Ethnic Group	Brain MRI
1	P1	c.532G>C p.(Ala178Pro) c.298-768T>C p.(?)	M	Normal pregnancy and delivery via C-section, no foetal distress	NO	8 m/ Hyposomia, mild hepatomegaly, non ketotic hypoglycemia, high lactate	Psychomotor delay, metabolic acidosis, Fanconi syndrome	Stable/20 y	European caucasian	Cavitating leukodystrophy (Supplemental Figure 1)
	P2		M	Normal pregnancy and delivery via C-section, foetal distress after few hours		Neonatal/Severe metabolic acidosis	NA	Metabolic derangement/death 6 m		NA
2	P3	c.485del p.(Asn162Ilefs*27) c.420+784C>T p.(?)	M	Normal pregnancy and delivery, foetal distress	NO	30 m/Frequent falls	Bradykinesia and limbs dystonia	Wheelchair bound, severe dysarthria/18 y	European caucasian	Bilateral T2 hyperintensity of the caudate and putamen and left dentate involvement with focal necrosis
	P4		M	Normal pregnancy and delivery, no foetal distress		4 y/Dystonia, decreased fine motor skills	Bradykinesia and limbs dystonia	Wheelchair bound, severe dysarthria/13 y		Bilateral striatal necrosis
3	P5	c.967del p.(Tyr323Ilefs*18) c.420+784C>T p.?	M	Normal pregnancy and delivery, no foetal distress	NO	2 y/Speech delay, psychomotor regression, with loss of ambulation	Spastic-dystonic tetraparesis	Stable/5 y	European caucasian	Bilateral symmetric T2 hyperintensity in basal ganglia, brainstem, cerebellar dentate nuclei, right thalamus, and periaqueductal gray
4	P6	c.2T>C p.(Met1?) c.532G>C p.(Ala178Pro)	M	Normal pregnancy and delivery, no foetal distress	NO	2 y/Left hemiparesis and dystonia	Neurological regression, loss of walking, sitting and head control (2,2 y)	Respiratory distress in a context of inhalation pneumonia /12 y deceased	European caucasian	Bilateral and symmetric T2 hyperintensity of basal ganglia
5	P7	c.337C>T p.(Arg113*) c.420+784C>T p.?	M	Normal pregnancy and delivery, no foetal distress	NO	3 y/Frequent falls, dysarthria.	Gait instability, frequent falls, dystonia	Stable/25 y	North African caucasian	Bilateral and symmetric hyperintensity in T2-weighted images of the putamina and caudate nuclei.
	P8		F	Normal pregnancy and delivery, no foetal distress		5 y/Frequent falls, dysarthria.	At 10 y: oss of walking, dysarthria, dystonia and pyramidal signs	Stable/24 y		Bilateral and symmetric hyperintensity in T2-weighted images of the putamina and caudate nuclei.

	P9		F	Normal pregnancy and delivery, no foetal distress		5 y/ Mild motor and language delayed.	Dysarthria, axial hypotonia, loss of walking and spastic-dystonic tetraparesis	Stable/20 y		Bilateral basal ganglia T2 hyperintensity
6	P10	c.532G>C p.(Ala178Pro)<br><br>c.536A>G p.(Glu179Gly)	F	Normal pregnancy and delivery, no foetal distress, normal birth parameters	NO	2 m/Hypotonia, poor contact, lactic acidosis	NA	NA/3 m deceased	European caucasian	Bilateral striatal necrosis and involvement of white matter
7	P11	c.237del p.(Pro80Leufs*11)<br><br>c.920A>G p.(Asp307Gly)	F	Normal pregnancy and delivery, no foetal distress	NO	5 w/Dysphagia, hypotonia, lack of contact, peripheral hypertonia, epilepsy	Hypotonia, lack of contact, pharmaco-R epilepsy	NA/6 w deceased	European caucasian	Bilateral basal ganglia T2 hyperintensity
8	P12	c.266C>T p.(Ala89Val)	F	Normal pregnancy and delivery, no foetal distress	YES	3 y/ Psychomotor regression and hypotonia; dystonic movements	Hypotonia, peripheral neuropathy	Severe global hypotonia with dystonic movements; speech delay/10 y	North African caucasian	Symmetrical basal ganglia T2 hyperintensity
9	P13	c.420+784C>T p.?<br><br>c.532G>C p.(Ala178Pro)	M	Normal pregnancy and delivery, no foetal distress	NO	3 y/Frequent falls, dystonia spasticity, dysarthria	NA	Currently stable disease course/10 y	North African caucasian	Bilateral basal ganglia T2-hyperintensity involving putamen, lenticular and caudate nuclei.
10	P14	c.226T>C p.(Ser76Pro)	M	Consanguinity; Normal pregnancy delivery via C-section	YES	9 m/Developmental regression; hypotonia; axial weakness; feeding difficulties	Motor impairment, dysphagia, central hypotonia, peripheral spasticity, dystonia	Peripheral tone gradually increased, severe dysphonia that required an electronic communication aid/21 y	Sudanese	Bilateral basal ganglia and brainstem involvement
	P15		M	Consanguinity; normal pregnancy and delivery via C-section		14 m/Abnormal left foot posture	Gross motor delay, axial hypotonia, dysphonia/aphonia, dysphagia	Limited ability to move on his own; gastrostomy feeding/9 y deceased		Bilateral and symmetrical abnormal signal changes in the putamina, parietal white matter, and dorsal pons
11	P16	c.581-7A>G p.(Leu193_Gly194 insValIle)<br><br>c.805C>T p.(His269Tyr)	F	NA	NA	Developmental delay; dyskinetic movements	NA	NA/10 y	White Irish	Bilateral striatal necrosis and involvement of grey and white matter
12	P17	c.659C>A p.(Thr220Lys)	F	Born at term in good conditions	NA	18 h/Severe metabolic acidosi which persisted through life	NA	NA/9 w deceased	Pakistani	NA
	P18		F	NA		NA	NA	NA		NA

13	P19	c.659C>A p.(Thr220Lys)	F	Born in good conditions	NA	3 m/Admitted in the paediatric intensive care unit for increased sleepiness secondary to lactic acidosis prior to demise	NA	NA/3 m deceased	Pakistani	NA
	P20 Aunt of P19		F	NA	NA	NA	NA	NA/ deceased		NA
	P21 Uncle of P19		M	NA	NA	NA	NA	NA/ deceased		NA
14	P22	c.805C>T p.(His269Tyr)	M	Born at term after a normal pregnancy	YES	3 m/Axial hypotonia, peripheral hypertonia, limited contact, lactic acidosis	Axial hypotonia, peripheral hypertonia, convulsive movements	At 8 m improvement in neurodevelopment al milestones/ 9 m deceased for pneumonia	Turkish	Bilateral basal ganglia T2 hyperintensity and cortical hyperintensity
15	P23	c.532G>C p.(Ala178Pro)	F	Born at 38 GW by emergency C-section,asphyxia, lactic acidosis at 2nd day of life – good response to DCA	YES	After birth/Severe growth delay at birth, severe lactic acidosis,hypertonicity, proximal renal tubular acidosis	At 6 m: hypertonicity, optic atrophy	Severe neurodevelopment al and psychomotor delay/4 y deceased	European Caucasian	Deep cerebral and cerebellar white matter and basal ganglia hyperintensity
16	P24	c.206A>T p.(Asp69Val)	F	Born at 32 GW by emergency C-section hospitalization for prematurity and feeding difficulty	YES	11 m/Severe delay, lactic acidosis, seizures, hypertonicity, hypertrophic cardiomyopathy	Severe neuromotor retardation, hypertonicity	Severe neuromotor retardation, recurrent lactic acidosis attacks/18 m deceased	Middle Easterners Caucasian	Abnormal T2 signal hyperintensity in periventricular white matter, external capsule, corpus callosum, and thalami.
	P25		M	39 GW, preimplantation genetic diagnosis due to sibling, healthy at birth		26 m/Preimplantation genetic diagnosis due to sibling. No symptom on diagnosis	Mild neurodevelopmental delay, mild hypotonicity, ataxic gait	Mild psychomotor delay, proximal renal tubular acidosis/3 y		Signal abnormalities involving DWM, optic nerve and cervical spine
17	P26	c.820A>G Homozygous p.(Arg274Gly)	F	33 GW, by spontaneous vaginal delivery hospitalization due to dysmorphic features and hypotonicity	NA	5 y/Severe neuromotor delay and reduction of spontaneous movements	Truncal hypotonia, mild swallowing difficulties	Psychomotor delay, lactic acidosis/7.5 y	European Caucasian	Signal abnormality on corpus striatum, nucleus caudatus, globus pallidus, putamen, medial thalamic nucleus and brainstem
18	P27	c.230T>C p.(Leu77Pro) c.371T>C p.(Ile124Thr)	M	Born at 36 GW by C-section C-after normal pregnancy NICU 2 days with O2 application	NO	20 m/ Psychomotor delay, oral dystonia, axial hypotonia	Intact cranial nerves, dysarthria, dystonia, hypertonia of limbs, muscle weakness, asymmetrical gait	Delay (plataue course), became progressive during last 2 months/4 y	Middle East Caucasian	Bilateral hyperintense lesions involving putamen and globi pallidi

Abbreviations: h: hours; w: weeks; m: months; y: years; GW: gestational week; DWM: deep white matter; NA: not available; M: male; F: female.
NDUFAF6 RefSeq: NM_152416.4

Supplementary Table 2 Genetic findings

Patients	cDNA and protein change	Segregation	Genomic location (hg19/hg38)	Allele frequency gnomAD v2.1.1	Allele frequency gnomAD v4.1.0	ACMG/AMP Criteria	References
P1, P2	c.532G>C p.(Ala178Pro)	mother	Chr8(GRCh37):g.96057827 G>C	0.000112		**P:** PM2_supp, PP3_supp, PP5_str PS3_str	Catania A. et al., 2018
	c.298-768T>C p.(?)	father	Chr8(GRCh37):g.96046914 T>C	-		**LP:** PM3_mod, PM2_supp, PS3_str	Hartmannová H, et al., 2016
P3, P4	c.485del p.(Asn162Ilefs*27)	mother	Chr8(GRCh37):g.96057774 Adel	0.00000402		**P:** PVS1_vstr, PM2_supp, PP5_supp	Gedikbasi A. et al., 2023
	c.420+784C>T p.(?)	father	Chr8(GRCh37):g.96048588 C>T	0.0000468		**LP:** PP5_mod, PM2-supp, PS3str	Catania et al. 2018
P5	c.967del p.(Tyr323Ilefs*18)	father	Chr8(GRCh37):g.96070130 Tdel	0.0000282		**P:** PVS1_vstr, PM2_supp, PS3_str	Novel
	c.420+784C>T p.(?)	mother	Chr8(GRCh37):g.96048588 C>T	0.0000468		**LP:** PP5_mod, PM2-supp, PS3str	Catania et al. 2018
P6	c.2T>C p.Met1?	mother	Chr8(GRCh37):g.96037238 T>C	-		**P:** PM2_supp, PM3_mod, PP3_supp, PP5_str PS3_str	Novel
	c.532G>C p.(Ala178Pro)	?	Chr8(GRCh37):g.96057827 G>C	0.000112		**P:** PM2_supp, PP3_supp, PP5_str PS3_str	Catania et al. 2018
P7, P8, P9	c.337C>T p.(Arg113*)	father	Chr8(GRCh37):g.96047721 C>T	0.000016		**P:** PVS1_vstr, PM2_supp, PP5_supp	Hu C. et al., 2020
	c.420+784C>T p.(?)	mother					Catania et al. 2018
P10	c.536A>G p.(Glu179Gly)	mother	Chr8(GRCh37):g.96057831 A>G	0.000016		**P:** PM2_supp, PP3_supp, PP5_str PS3_str	Novel

	c.532G>C<br>p.(Ala178Pro)	father	Chr8(GRCh37):g.96057827<br>G>C	0.000112		**P:** PM2_supp,<br>PP3_supp,<br>PP5_str<br>PS3_str	Catania et al. 2018
P11	c.237del<br>p.(Pro80Leufs*11)	father	Chr8(GRCh37):g.96044262<br>Cdel	0.00000398		**LP:**<br>PVS1_vstr,<br>PM2_supp	Novel
	c.920A>G<br>p.(Asp307Gly)	mother	Chr8(GRCh37):g.96070083<br>A>G	-		**LP:** PS3_mod;<br>PM2_mod;<br>PM3_mod;<br>PP4_sup	Novel
P12	Homozygous<br>c.266C>T<br>p.(Ala89Val)	mother/?	Chr8(GRCh37):g.96044291<br>C>T	-		**LP:** PP3_str,<br>PM2-supp,<br>PS3_str	Novel
P13	c.420+784C>T<br>p.?	father	Chr8(GRCh37):g.96048588<br>C>T	0.0000468		**LP:** PP5_mod,<br>PM2-supp,<br>PS3str	Catania et al. 2018
	c.532G>C<br>p.(Ala178Pro)	mother	Chr8(GRCh37):g.96057827<br>G>C	0.000112		**P:** PM2_supp,<br>PP3_supp,<br>PP5_str<br>PS3_str	Catania et al. 2018
P14, P15	Homozygous<br>c.226T>C<br>p.(Ser76Pro)	father<br>mother	Chr8(GRCh38):g.95032023<br>T>C		$6.2x10^{-6}$	**LP:** PP3_mod,<br>PM2_supp,<br>PP5_supp,<br>PS3_mod	Kohda M. et al.,<br>2016
P16	c.581-7A>G<br>p.(Leu193_Gly194<br>insValIle)	mother	Chr8(GRCh38):g.95046987<br>A>G		0.00003098	**LP:** PP3_str,<br>PM2_supp,<br>PP3_mod	Novel
	c.805C>T<br>p.(His269Tyr)	?	Chr8(GRCh38):g.95048547<br>C>T		0.000003099	**LP:** PP3_str,<br>PM2_supp,<br>PS3_str	Kohda M. et al.,<br>2016
P17, P18,<br>P19, P20,<br>P21	Homozygous<br>c.659C>A<br>p.(Thr220Lys)	father<br>mother	Chr8(GRCh38):g.95047072<br>C>A		0.000001239	**LP:**<br>PM2_supp,<br>PS3_str,<br>PP1_supp	Novel
P22	Homozygous<br>c.805C>T<br>p.(His269Tyr)	father<br>mother	Chr8(GRCh38):g.95048547<br>C>T		0.000003099	**LP:** PP3_str,<br>PM2_supp,<br>PS3_str	Kohda M. et al.,<br>2016
P23	Homozygous<br>c.532G>C<br>p.(Ala178Pro)	father<br>mother	Chr8(GRCh37):g.96057827<br>G>C	0.000112		**P:** PM2_supp,<br>PP3_supp,<br>PP5_str<br>PS3_str	Catania et al. 2018

P24, P25	Homozygous c.206A>T p.(Asp69Val)	father mother	Chr8(GRCh38):g.95032003 A>T		0.000001859	**LP:** PP3_str, PM2_supp, PP5_supp	Kohda M. et al., 2016
P26	Homozygous c.820A>G p.(Arg274Gly)	father mother	Chr8(GRCh38):g.95052177 A>G		0.000001239	**LP:** PP3_str, PM2_supp, PP5_supp, PS3_mod	Kohda M. et al., 2016
P27	c.230T>C p.(Leu77Pro)	mother	Chr8(GRCh37):g.96044255 T>C	0.0000922		**LP:** PM3_mod, PM2_supp, PP3_str	Novel
	c.371T>C p.(Ile124Thr)	father	Chr8(GRCh37):g.96047755 T>C	0.0000922		**LP:** PS3_str, PM2_supp, PP5_supp	Kohda M. et al., 2016

Supplementary Table 3 Clinical and MRI features from reported cases carrying variants in *NDUFAF6*

Patients	Gender	NDUFAF6 variants	Consanguinity/ Etnicity	First symptoms/ Onset age	Neurological signs progression/Age at last examination	MRI findings	CI activity/ Organic acids	References
P1	M	c.371T>C (p.Ile308Thr)<br>c.923T>C (p.Asp307Val)	NA/Asiatic (?)	Walking instability/3y and 7 m	Motor regression and language regression/6y and 3m	T2WI: Bilateral symmetric hyperintense lesions in the putamen.<br>T1WI: Hypointense lesions in the same regions.	NA/Lactate: H	Zhou Y. *et al.*, 2024
P2	M	c.371T>C (p.Ile308Thr)<br>c.920A>T (p.Asp307Val)	NA/Asiatic (?)	Developmental delay and ataxic gait/2 **y**	Unsteady walking, lack of coordination, speech developmental delay/3y	T2WI: Bilateral symmetric hyperintense lesions in the putamen.<br>T1WI: Hypointense lesions in the same regions.	NA/Lactate: H	
Single case	M	c.371T > C (p.Ile124Thr)<br>c.233_242del (p.Leu78GInfs*10)	NO/NA	Developmental and speech delay/4.6 y	Dystonic and ataxic movements/5.4y	Signals in the bilateral putamen, lesions of symmetric basal ganglia	Reduced/Lactate: H	Kim J. *et al.*, 2022
2 siblings	M	c.371T>C (p.Ile124Thr)<br>c.420+2_420+3insTA (p.?)	NO/Caucasian	Tiptoeing and increasingly severe focal hand dystonia/4y	Generalized dystonia and normal cognitive abilities stable over time/27y	Bilateral T2 hyperintensities in the putamen; no WM abnormalities.	Normal/Lactate and pyruvate: N	Johnstone T. *et al.*, 2020
	F	c.371T>C (p.Ile124Thr)<br>c.420+2_420+3insTA (p.?)		Tiptoeing and increasingly severe focal hand dystonia/4y	Generalized dystonia/NA	NA	NA	
3 siblings: P1	NA	c.554_558delTTCTT (p.Tyr187AsnfsTer65)<br>c.371T>C (p.Ile124Thr)	NO/Caucasian	Toe walking and speech difficulties/between 17m and 2.5 y	Generalized dystonia and gait loss; speech difficulties; dysphagia/11y	T2 hyper-intensity in the putame; volume loss in the putamen, caudate, pallidum or subthalamus.	Reduced/Lactate: N	Baide-Mairena H. *et al.*, 2019
P2	NA			Toe walking and speech difficulties/between 17m and 2.5 y	Generalized dystonia and gait loss; speech difficulties; dysphagia/8y	T2 hyper-intensity in the putame	Normal/Lactate: N	
P3	NA			Toe walking and speech difficulties/between 17m and 2.5 y	Generalized dystonia and gait loss; speech difficulties; dysphagia/6y	T2 hyper-intensity in the putamen and apparently normal volume	NA/NA	
2 siblings: A1	M	c.532G>C (p.Ala178Pro)<br>c.420+784C>T (p.?)	NO/Caucasian	Psychomotor regression/21 m	Slowly progressive drooling, dysarthria, dysmetria, tremor, severe ataxic gait and hypertonia. Normal cognitive function/5y	T2-hyperintensities in putamina, dentate nuclei and superior cerebellar peduncles and caudate nuclei	Reduced in fibroblasts and muscle/Lactate and pyruvate: N	Catania A. *et al.*, 2018
A2	F			Psychomotor regression/12 m	Limb dysmetria and trunk titubation; normal cognitive functions; autonomous gait at 24 months with ataxic features /3y	Bilateral T2-hyperintensities in dentate nucleus and superior cerebellar peduncle.	NA/Slightly increased lactate and pyruvate	
B	M	c.532G>C (p.Ala178Pro)<br>c.420+784C>T (p.?)	NO/Caucasian	Motor and language disturbances/3.5y	Severe gait impairment, dysarthria and early occurrence of dystonic movements.	Necrotic damage of the putamen, gradually extending to dentate nuclei and anterior caudate nuclei.	Reduced in fibroblasts; normal in muscle/Lactate and pyruvate: N	
C	M	c.532G>C (p.Ala178Pro)<br>c.420+784C>T (p.?)	NO/Caucasian	Gait unsteadiness and motor coordination problems/5y	Extrapyramidal syndrome; cognitive functions were preserved/11y	Bilateral T2-hyperintense lesions of the putamen	Normal in muscle/Lactate and pyruvate: N	

9 affected individuals	M/F	c.298-768T>C (p.?) homozygous	Acadian population/Caucasian	Genu valgum/before 10y	Fanconi syndrome	NA	Reduced/Lactate: H	Hartmannová H. *et al.*, 2016
2 siblings: P1	F	c.296A>G (p.Gln99Arg) homozygous	Yes/ Levantine Middle Eastern	Focal right-hand seizures/10m	Ataxia, rigidity, decreased movement and strength; Cardiorespiratory impairment/34m deceased	Basal ganglia necrosis of the caudate and putamen/22m	Reduced in fibroblasts, muscle and liver/Lactate: H	Pagliarini D.J. *et al.*, 2008
P2	M			Focal right-hand seizures/7m	Ataxia, rigidity, decreased movement and strength /22m	Basal ganglia necrosis of the caudate and putamen	Reduced in fibroblasts/ Lactate: H	
2 siblings: P9	M	c.226T > C (p.Ser76Pro) homozygous	NA/NA	NA	Generalized dystonia/NA	T2 bilateral changes in the putamen, parietal cerebral WM, dorsal pons	Reduced/NA	Martikainen M.H. *et al.*, 2016
P10	M			NA	Generalized dystonia/NA	T2 bilateral changes in the caudate and putamen nucleus.	Reduced/NA	

M: male; F: female; y: years; m: months; T2WI: T2-weighted imaging; T1WI: T1-weighted imaging; NA: Not Available; WM: white matter; H: high, L: low; N: normal
All NDUDAF6 cases described in Khoda et al lacked clinical informations.

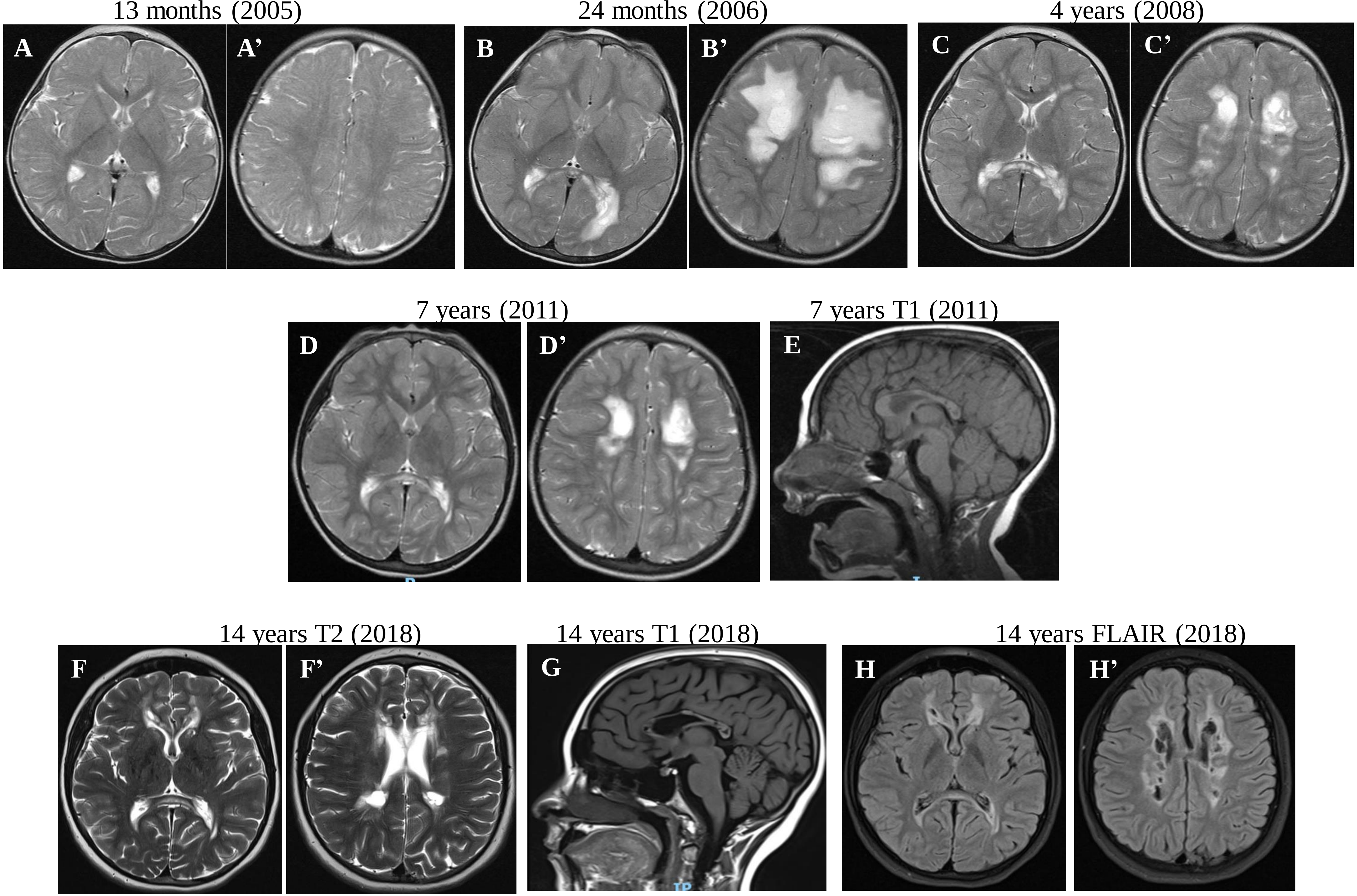
13 months (2005)
24 months (2006)
4 years (2008)
A
A'
B
B'
C
C'
7 years (2011)
7 years T1 (2011)
D
D'
E
14 years T2 (2018)
14 years T1 (2018)
14 years FLAIR (2018)
F
F'
G
H
H'

Supplementary Figure 1 Neuroimaging study of P1. Serial brain MRI images of P1 performed at age 13 months (A, A'), 24 months (B, B'), 4 years (C, C'), 7 years (D, D', E), and 14 years (F-H'). Figures A-D', and F-F' are T2 weighted images; Figures E, and G are T1 weighted images, and Figures H, H' are FLAIR images. Notice a normal MRI at age 13 months, and formation of white matter cavitations at age 24 months, that were confirmed until age 14 years; cavitations were also found at the level of the corpus callosum (E, G).

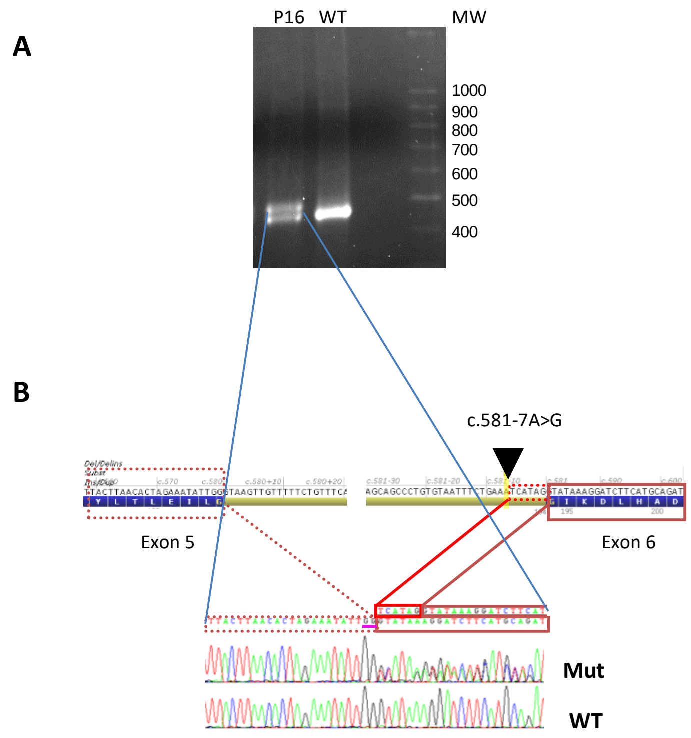

Supplementary Figure 2 mRNA evaluation in P16. (A) Gel electrophoresis showing an additional higher molecular weight band in P16. RNA was extracted from patient and control fibroblasts using the SV Total RNA Isolation System (Promega), whilst the Promega M-MLV reverse transcriptase was used for first strand synthesis. (B) Electropherograms and graphical representation of the c.581-7A>G impact at mRNA level. Mut: Mutant; WT: wild-type; P16: Patient 16; MW: molecular weight.

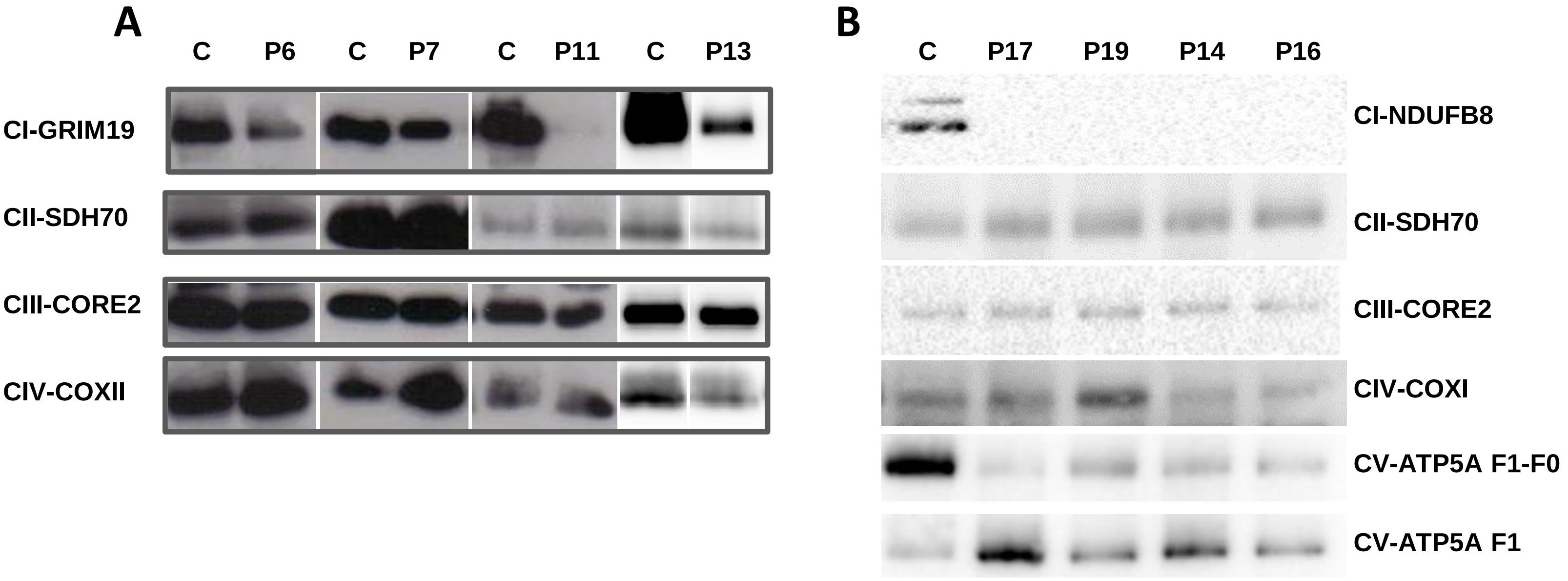

Supplementary Figure 3 BN-PAGE of the assembly status of CI. Blue native polyacrylamide gel electrophoresis was performed on mitoplasts isolated from fibroblasts of (A) P6, P7, P11, P13 and (B) P14, P16, P17, P19 and probed with antibodies conjugated against subunits of OXPHOS complexes. Experiments were performed on two groups of patients across different laboratories (P6, P7, P11, P13, and P14, P16, P17, P19 respectively). P6, P7, P11 and P13 were run on different gels.

Supplementary File 1. Cropped and uncropped blots of NDUFAF6 protein presented in Fig. 3A

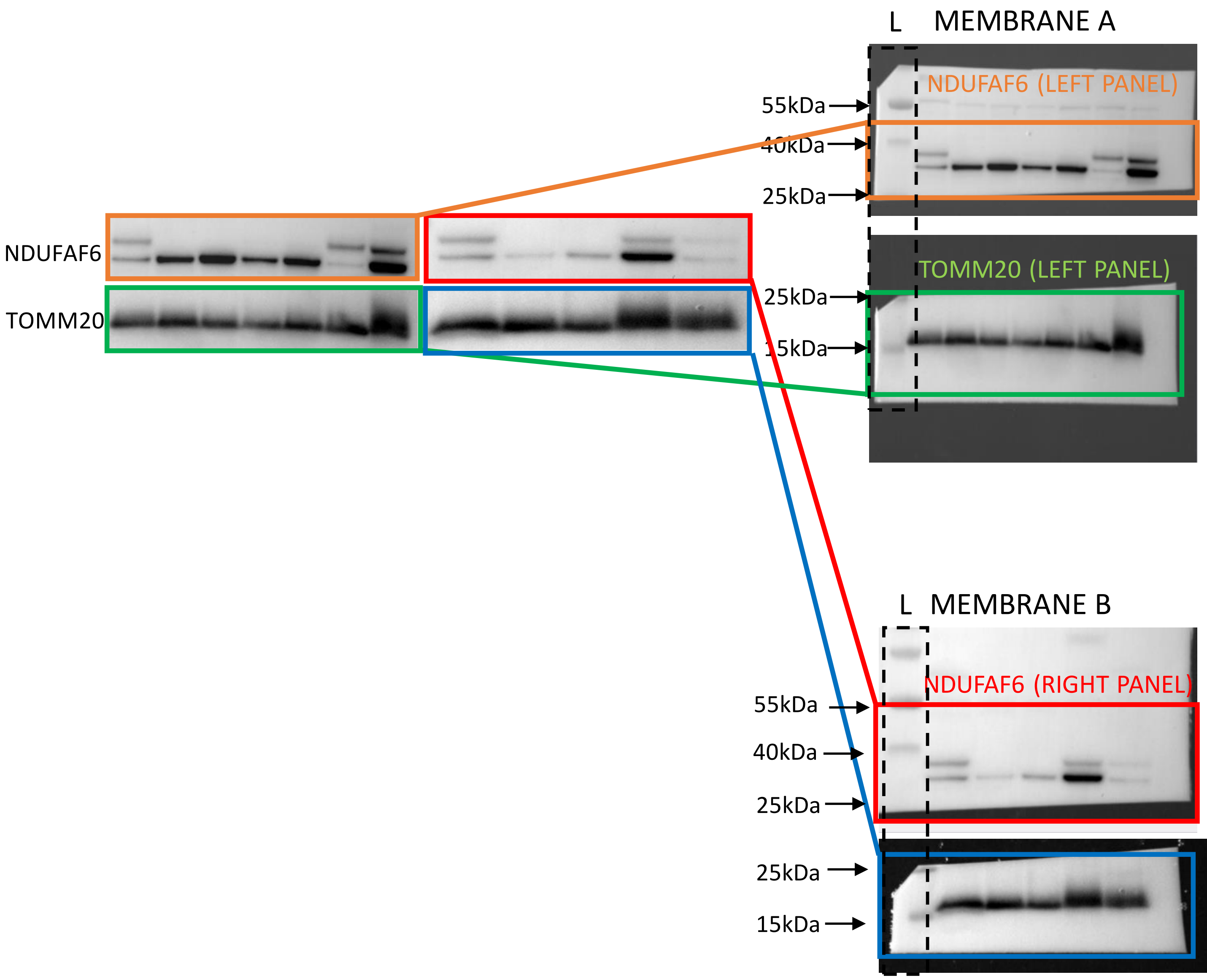

Legend: Western blotting of SDS-Page represented in Figure 3A of the main text. To allow the contemporary blotting of multiple antibodies, both membranes (A and B) were cut at 25kDa and blotted with NDUFAF6 (upper part) and TOMM20 (lower part). TOMM20 was used for loading normalization. L: ladder.

Supplementary File 2. Cropped and uncropped blots of CI subunits presented in Fig. 3B

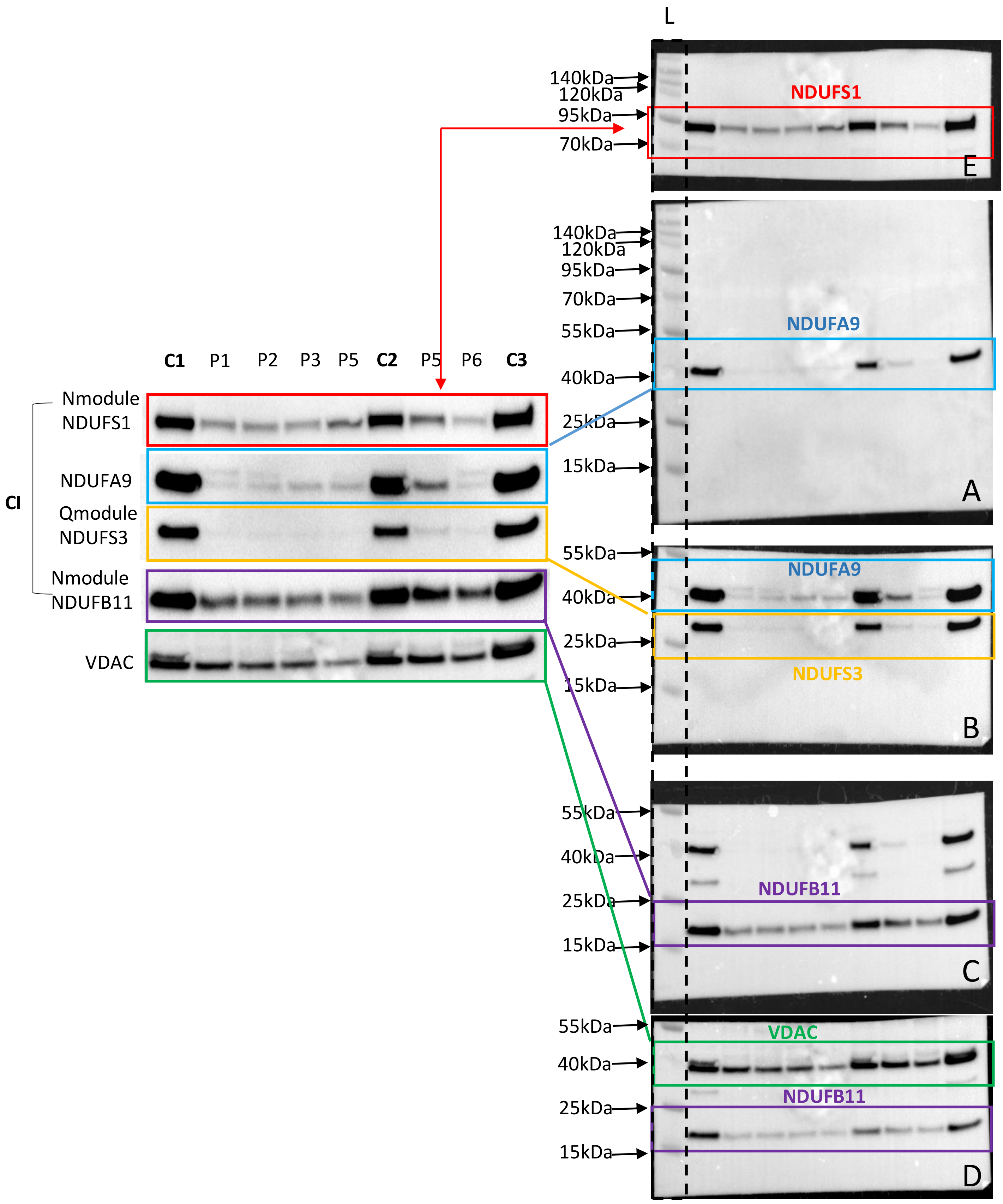

Legend: Western blotting of SDS-Page represented in Figure 3B of the main text. Membrane A was initially blotted with NDUFA9 antibody (A). Subsequently the membrane was cut at 55kDa and the lower part was blotted with NDUFS3 (B), NDUFB11 (C) and VDAC (D) antibodies; the upper membrane was blotted with NDUFS1 (E) antibody. VDAC was used to assess equal protein loading. L: ladder

Supplementary File 3. Cropped and uncropped blots of other OXPHOS subunits presented in Fig. 3B

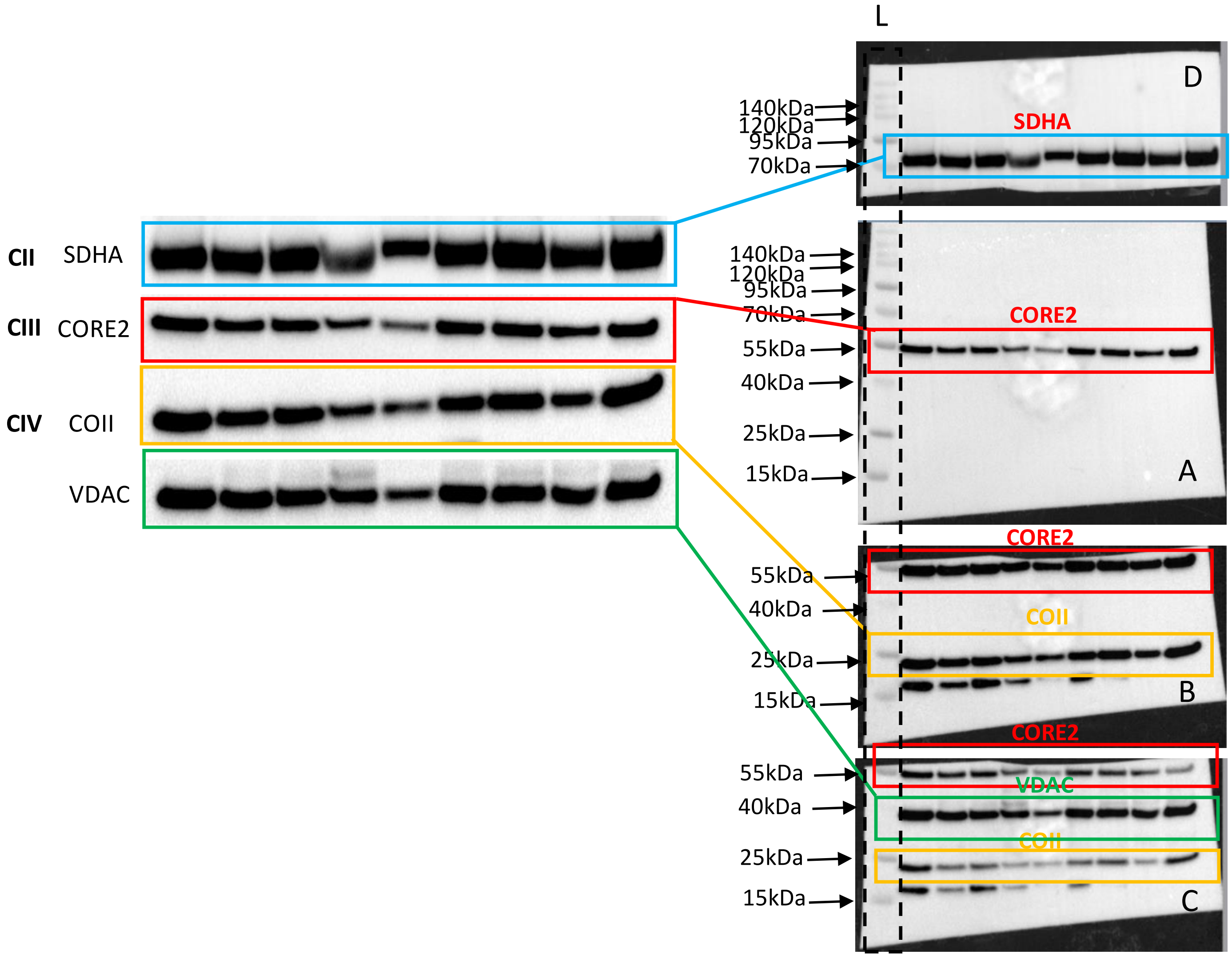

Legend: Western blotting of SDS-Page shown in Figure 3B of the main text. Membrane A was initially probed with CORE2 antibody. Subsequently the membrane was cut just above 55kDa and the lower part was probed with COII (B) and VDAC (C) antibodies; the upper membrane was probed with SDHA (D) antibody. VDAC antibody was used as a control of equal loading. L: ladder.

Supplementary File 4. Cropped and uncropped images of IGA of CI and of western blotting of the proteins used as equal loading (Fig. 4A)

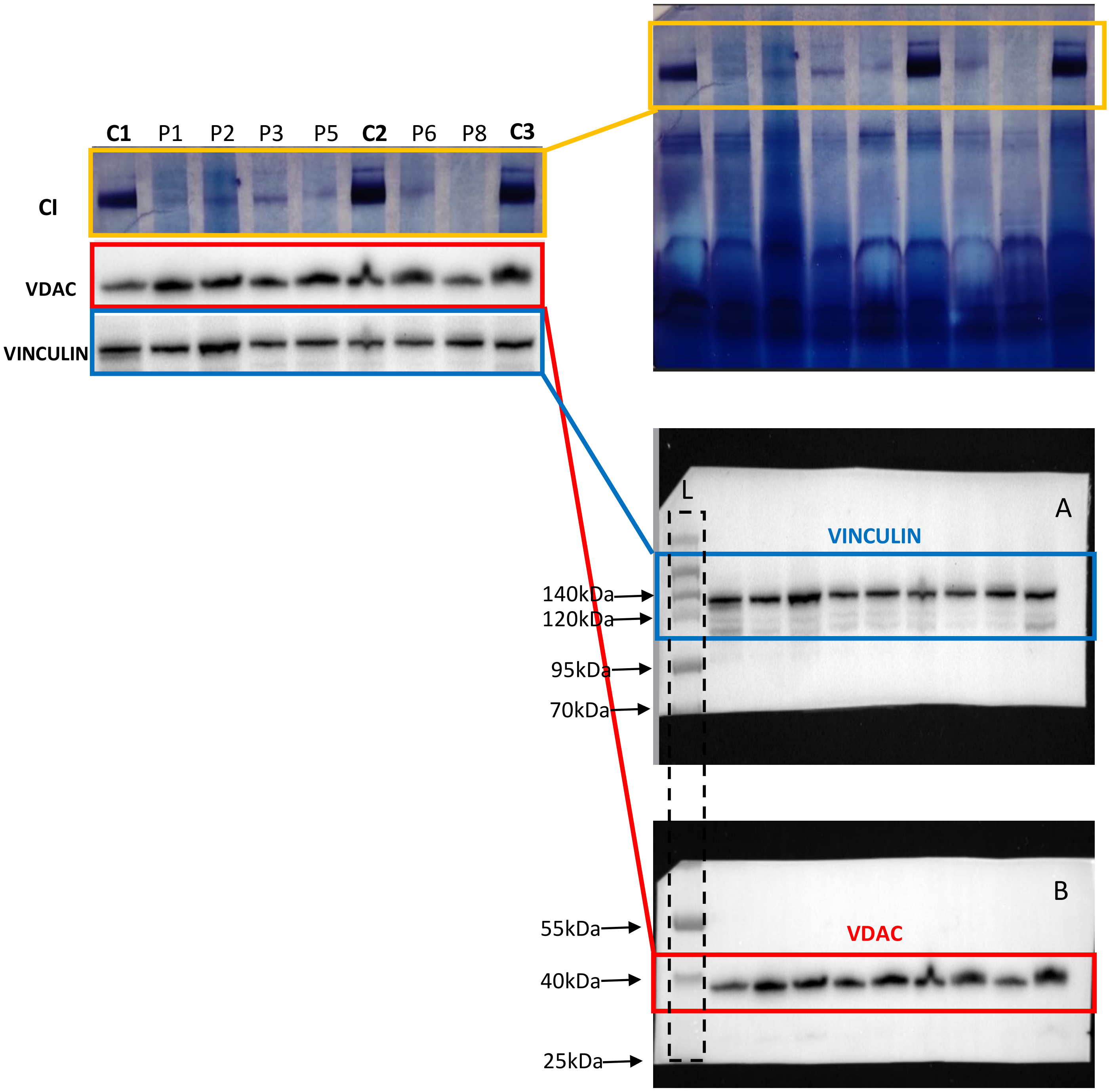

Legend: *In-gel* activity assay performed on blue native gels and western blotting performed on SDS-page as shown in Figure 4A of the main text. Membranes A and B originate from the same membrane, which was cut at 70 kDa and probed with vinculin (upper membrane) and VDAC (lower membrane). Vinculin and VDAC antibodies were used as loading controls. L: molecular weight ladder.

Supplementary File 5. Cropped and uncropped blots of supercomplexes presented in Fig. 4C

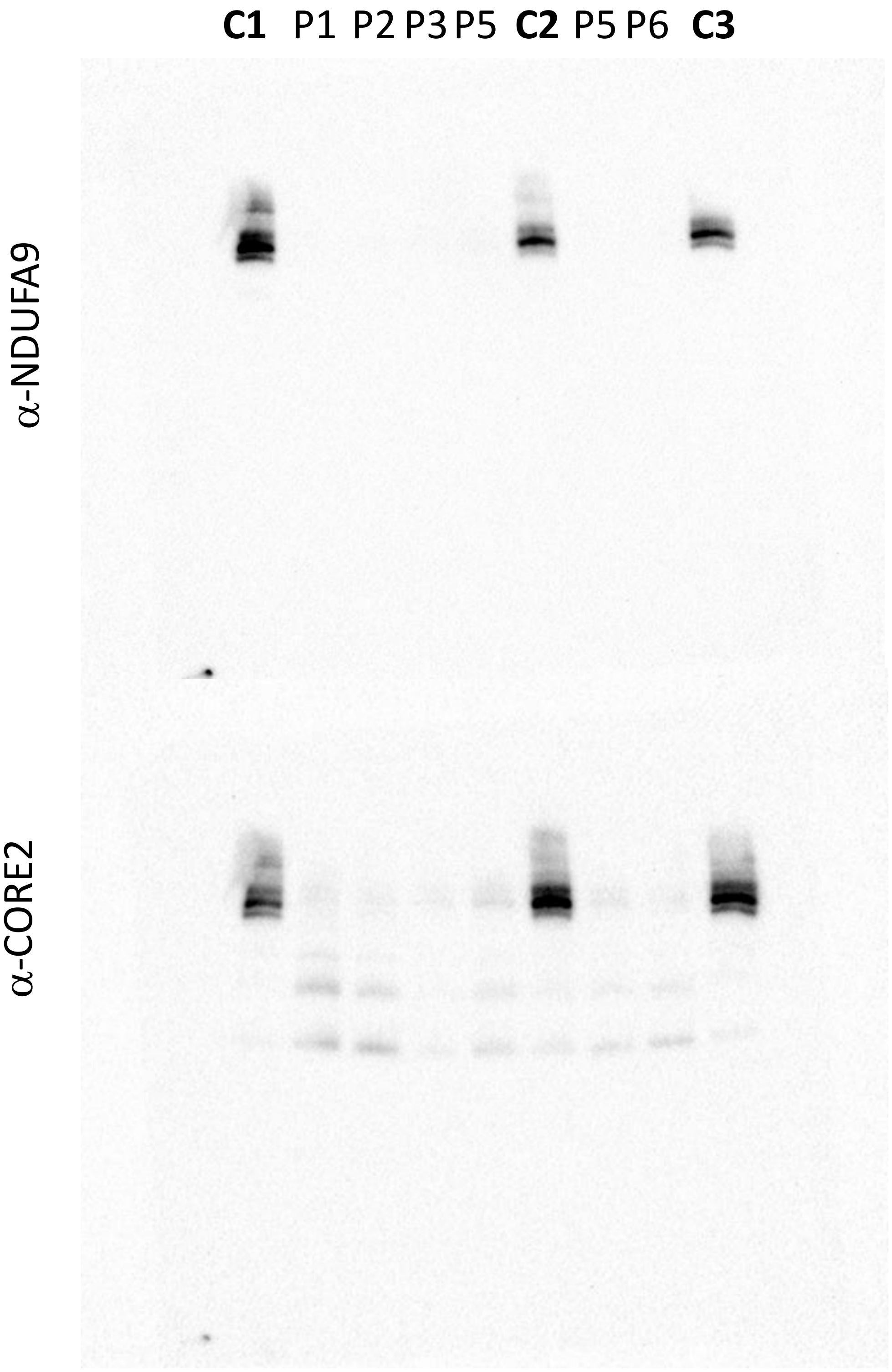

Legend: Blue native gel subjected to western blotting as shown in Figure 4C of the manuscript and blotted first with NDUFA9 antibody and subsequently with CORE2 antibody. Figure 4B of the main text is already an uncropped image of the blue native gel.
